# XAB2 promotes Ku eviction from single-ended DNA double-strand breaks independently of the ATM kinase

**DOI:** 10.1093/nar/gkab785

**Published:** 2021-09-09

**Authors:** Abhishek Bharadwaj Sharma, Hélène Erasimus, Lia Pinto, Marie-Christine Caron, Diyavarshini Gopaul, Thibaut Peterlini, Katrin Neumann, Petr V Nazarov, Sabrina Fritah, Barbara Klink, Christel C Herold-Mende, Simone P Niclou, Philippe Pasero, Patrick Calsou, Jean-Yves Masson, Sébastien Britton, Eric Van Dyck

**Affiliations:** DNA Repair and Chemoresistance Group, Department of Oncology, Luxembourg Institute of Health (LIH), Luxembourg, Luxembourg; DNA Repair and Chemoresistance Group, Department of Oncology, Luxembourg Institute of Health (LIH), Luxembourg, Luxembourg; Faculty of Science, Technology and Communication, University of Luxembourg, Esch-sur-Alzette, Luxembourg; DNA Repair and Chemoresistance Group, Department of Oncology, Luxembourg Institute of Health (LIH), Luxembourg, Luxembourg; Faculty of Science, Technology and Communication, University of Luxembourg, Esch-sur-Alzette, Luxembourg; CHU de Québec Research Center, Oncology Division, Québec City, Canada; Department of Molecular Biology, Medical Biochemistry and Pathology, Laval University Cancer Research Center, Québec City, Canada; Institut de Génétique Humaine, CNRS et Université de Montpellier, Equipe Labellisée Ligue Contre le Cancer, Montpellier, France; CHU de Québec Research Center, Oncology Division, Québec City, Canada; Department of Molecular Biology, Medical Biochemistry and Pathology, Laval University Cancer Research Center, Québec City, Canada; DNA Repair and Chemoresistance Group, Department of Oncology, Luxembourg Institute of Health (LIH), Luxembourg, Luxembourg; Quantitative Biology Unit, Multiomics Data Science Group, LIH, Luxembourg; NorLux Neuro-Oncology Laboratory, Department of Oncology, LIH, Luxembourg; National Center of Genetics, Laboratoire National de Santé, Dudelange, Luxembourg; Functional Tumour Genetics Group, Department of Oncology, LIH, Luxembourg; Department of Neurosurgery, University Clinic Heidelberg, Heidelberg, Germany; NorLux Neuro-Oncology Laboratory, Department of Oncology, LIH, Luxembourg; Department of Biomedicine, University of Bergen, Norway; Institut de Génétique Humaine, CNRS et Université de Montpellier, Equipe Labellisée Ligue Contre le Cancer, Montpellier, France; Institut de Pharmacologie et de Biologie Structurale, Université de Toulouse, CNRS, UPS, Toulouse, France, Equipe Labellisée Ligue Nationale Contre le Cancer 2018; CHU de Québec Research Center, Oncology Division, Québec City, Canada; Department of Molecular Biology, Medical Biochemistry and Pathology, Laval University Cancer Research Center, Québec City, Canada; Institut de Pharmacologie et de Biologie Structurale, Université de Toulouse, CNRS, UPS, Toulouse, France, Equipe Labellisée Ligue Nationale Contre le Cancer 2018; DNA Repair and Chemoresistance Group, Department of Oncology, Luxembourg Institute of Health (LIH), Luxembourg, Luxembourg

## Abstract

Replication-associated single-ended DNA double-strand breaks (seDSBs) are repaired predominantly through RAD51-mediated homologous recombination (HR). Removal of the non-homologous end-joining (NHEJ) factor Ku from resected seDSB ends is crucial for HR. The coordinated actions of MRE11-CtIP nuclease activities orchestrated by ATM define one pathway for Ku eviction. Here, we identify the pre-mRNA splicing protein XAB2 as a factor required for resistance to seDSBs induced by the chemotherapeutic alkylator temozolomide. Moreover, we show that XAB2 prevents Ku retention and abortive HR at seDSBs induced by temozolomide and camptothecin, via a pathway that operates in parallel to the ATM-CtIP-MRE11 axis. Although XAB2 depletion preserved RAD51 focus formation, the resulting RAD51-ssDNA associations were unproductive, leading to increased NHEJ engagement in S/G2 and genetic instability. Overexpression of RAD51 or RAD52 rescued the XAB2 defects and XAB2 loss was synthetically lethal with RAD52 inhibition, providing potential perspectives in cancer therapy.

## Graphical Abstract

**Graphical Abstract:**
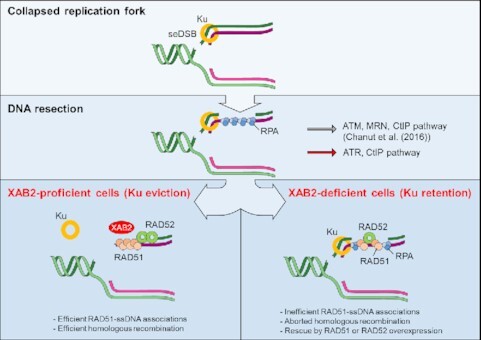
XAB2 promotes Ku eviction from single-ended DNA double-strand breaks independently of the ATM kinase.

## INTRODUCTION

DNA double-strand breaks (DSBs) represent the most lethal form of DNA lesions induced by ionizing radiation and chemotherapeutic genotoxicants (1). A special form of DSB called single-ended DSB (seDSB) occurs when the replication fork collapses upon encounter with single-strand DNA (ssDNA), base damage (2) or protein-DNA complexes such as those trapped by the topoisomerase I poison camptothecin (CPT) (3) or the poly(ADP-ribose) polymerase 1 (PARP1) inhibitor olaparib (4). Repair of these seDSBs is essential to maintain genetic integrity (5). As for two-ended DSBs (e.g. those induced by ionizing radiation (IR)), seDSBs can be processed by homology-directed or end-joining mechanisms. Homologous recombination (HR) mediated by the RAD51 recombinase plays a central role in replication fork repair during the S and G2 phases of the cell cycle in mammalian cells (6) through a recombination-dependent DNA replication pathway called break-induced replication (BIR) (7). Processing of seDSBs by non-homologous end-joining (NHEJ) is a toxic mechanism as it involves the juxtaposition and ligation of distant DNA ends, resulting in chromosomal aberrations and genetic instability (8). Unlike HR, which is restricted to the S and G2 phases of the cell cycle, NHEJ is active throughout interphase, including G1. In addition, seDSBs termini are initially sequestered by the DNA end-binding heterodimer Ku, a crucial NHEJ factor (9). Ku binding at seDSBs promotes NHEJ (10) and impairs RAD51-mediated HR (11). However, fully active HR outcompetes NHEJ in repairing seDSBs in S/G2, thus preventing genome instability (10).

During BIR, the seDSB is first resected to provide Replication Protein A (RPA)-coated, 3′ ssDNA overhangs on which RAD51 operates to replace RPA and assemble nucleoprotein filaments. These filaments mediate homology search and strand invasion into the homologous sister chromatid, generating a displacement loop (D-loop) (12). The single-strand annealing (SSA) factor RAD52 facilitates the assembly of ssDNA/RAD51 nucleoprotein filaments during HR-mediated seDSB repair in human cells (13). Similar to yeast RAD52 (12), human RAD52 can also promote BIR mechanisms without RAD51. Thus, in cancer cells undergoing replication stress, a BIR pathway has been described which critically depends on RAD52 (14). RAD52-mediated BIR also promotes mitosis DNA synthesis (MiDAS) at common fragile sites, a process where RAD51 is dispensable (15). RAD52 forms ring structures that interact with ssDNA, duplex DNA and DSB ends (16–18), exposing ssDNA at their outer surface (17,19,20). RAD52 rings catalyse the annealing of complementary strands generated during DSB end resection (21–23), as well as second-end capture in the repair of double-ended DSBs (24,25). Relevant to BIR, human RAD52 also promotes DNA strand exchange and D-loop formation in vitro (26–28).

DNA end resection occurs in the S and G2 phases of the cell cycle and involves the MRE11–RAD50–NBS1 (MRN) complex, C-terminal-binding protein interacting protein (CtIP), exonuclease 1 (EXO1), Bloom syndrome protein (BLM) and DNA2 nuclease/helicase (29,30). Resection is initiated at some distance from the seDSB by a nick introduced by the endonuclease activity of MRE11, itself activated by ataxia telangiectasia-mutated (ATM) kinase and CtIP. Bidirectional resection then takes place, mediated by MRE11 exonuclease activity in the 3′-5′ direction and EXO1/BLM/DNA2 in the opposite direction, generating ssDNA that recruits RPA. Although the mechanisms leading to the subsequent release of Ku remain obscure, for ∼40% of seDSBs induced by CPT, they involve the coordinated nuclease activities of MRE11 and CtIP, and activation by ATM (11). Regulation of end resection also involves p53 binding protein 1 (53BP1), effector molecules and the helicase HELB (30). Recently, several splicing factors have been involved in DNA end resection, including ZNF830 (31), Aquarius (32) and XAB2 (32,33), by mechanisms that remain to be elucidated.

The alkylating agent temozolomide is part of the standard of care for glioblastoma (GBM) patients (34). TMZ can induce seDSBs when its most cytotoxic lesion, *O*^6^-methylguanine (*O*^6^-meG), is left unrepaired by the DNA repair protein *O*^6^-methylguanine-DNA methyltransferase (MGMT) (35,36), whose gene promoter methylation represents a crucial clinical biomarker (37). Notably, MGMT defines a direct repair mechanism that reverses *O*^6^-meG lesions in a suicidal reaction resulting in the transfer of the methyl group to a cysteine residue in the active site of MGMT (35,36,38). Unrepaired *O*^6^-meG lesions generate *O*^6^-meG/thymidine mismatches during S phase. These mismatches are recognized, but not resolved, by the mismatch repair pathway, resulting in futile repair cycles and persistent single-stranded DNA (ssDNA) gaps that cause replication fork collapse and seDSBs (39–41). Several studies have underlined the involvement of HR in the repair of lesions resulting from *O*^6^-meG adducts (42,43).

Targeting DNA repair through inhibition of components of the DNA damage response (DDR) has emerged as an important therapeutic approach against many cancers (44). As a step to identify novel targets for the sensitization of GBM cells to TMZ, we carried out a shRNA screen for DDR genes that are required for cell proliferation in the presence of TMZ. Here, we report the characterization of XAB2, one of the top hits of this screen, and describe a novel role for XAB2 in promoting Ku eviction and HR at seDSBs, in parallel to the ATM-dependent pathway.

## MATERIALS AND METHODS

### Cell lines, cell cultures and treatments

NCH644 cells were kindly provided by Dr Christel Herold-Mende (Department of Neurosurgery, University of Heidelberg) (45). NCH644 cells were cultured in Neurobasal medium (Life Technologies, 21103049) supplemented with 1× B-27 (Life Technologies, 12587010) 2 mM l-glutamine, 20 U/ml Pen-Strep, 1 U/ml Heparin (Sigma-Aldrich, H3149-25KU), 20 ng/ml bFGF (Miltenyi, 130-093-841) and 20 ng/ml EGF (Provitro, 1325950500). For the shRNA screen, 2D cultures of NCH644 were carried out in the same medium using laminin-coated plates. U87 and U2OS cells were cultured in Dulbecco's modified Eagle's medium (Westburg, LO BE12-614F), supplemented with 10% fetal bovine serum (FBS) (Gibco, 10500-064), 50 U/ml Pen-Strep. Cells were routinely subjected to mycoplasma testing using the Mycoplasma PCR ELISA kit (Sigma-Aldrich, 11663925910) and tested negative.

Transductants and transfectants were selected using G418 (300 μg/ml) and/or puromycin (1 μg/ml). Stock solutions of Temozolomide (TMZ, 100 mM) (Sigma-Aldrich) and Camptothecin (CPT, 50 mM) (Sigma-Aldrich) were prepared in DMSO. RAD52 was inhibited using 6-hydroxy-dl-dopa (L-DOPA, Sigma-Aldrich) (46).

### shRNA screen, RNA interference and plasmids

The shRNA screen was performed as follows: NCH644 cells grown on laminin-coated plated were infected at an MOI of 0.3 with a 2.6 K custom lentiviral shRNA library constructed in pGIPZ puro vector and targeting 574 DNA Damage Response (DDR) genes (i.e. ∼4.5 shRNAs per gene on average, 700-fold representation)(Thermo Fisher Scientific). Cells were exposed to 1 μg/ml puromycin for 96 h to allow selection of transductants. Following harvesting of a reference sample (PD0), cells were split in two arms and cultured under vehicle (DMSO) or 60 μM TMZ (which corresponds to the IC20 for NCH644 interpolated from 2D cytotoxic assays and represents a sub-lethal, therapeutically-reachable dose range (47)). Cells were harvested after cumulative population doubling (PD) 8 and 15 for DNA extraction, library preparation and shRNA read counting via sequencing on an Illumina MiSeq platform. The screen was carried out in duplicate. Screen analysis (48) was performed by comparing shRNAs counts between the vehicle (DMSO) and TMZ treatment conditions using MAGeCK-RRA (49), ScreenBEAM (50) and HitSelect (51). For each PD, we crossed the lists of the top-15% candidate genes independently identified by the three algorithms and kept their intersection. We then considered as prioritized hits a subset of 26 genes consistently appearing in the 3 analyses both at PD8 and PD15 in both replicates.

shRNA-mediated depletion of XAB2 and RAD52 was carried out using pGIPZ (Dharmacon) or pLKO (52) lentiviral vector-based shRNAs. The following pGIPZ-based shRNAs were used: shXAB2-1 (V2LHS_50670; mature antisense: TTGACAGAGAATTGGTTCC), shXAB2-2 (V3LHS_645634; mature antisense: ACAAACGTAGCTGTATTGG), shRAD52-2 (V3LHS_376617; mature antisense: TCATGATATGAACCATCCT), shRAD52-3 (V2LHS_171209; mature antisense: ATTGCTTGAGGGCAAGGAG). Lentiviral vectors expressing non-silencing shRNAs in pGIPZ (puromycine resistance marker) (RHS4346) or in pLKO (neomycin resistance marker) (53) were used as negative controls.

siRNA-mediated depletion of XAB2 was achieved using SMARTpool siGENOME XAB2 siRNAs (Dharmacon), with siGENOME non-targeting control siRNA pool I used as a non-silencing siRNA control (Dharmacon). The sequences of the siRNAs targeting CtIP and MRE11 are reported in ref (11). siRNA transfections were carried out using Lipofectamine RNAiMAX transfection reagent, as detailed in (33).

Overexpression of MGMT, RAD51, RAD52, CtIP and MRE11: A Myc-tagged MGMT fragment containing 5′-XbaI extremities and 3′-BamHI extremities was generated by PCR amplification and restriction enzyme digestion and cloned into the lentiviral vector pCDH-EF1α-MCS-IRES-Neo and pCDH-EF1α-MCS-IRES-Puro (System Biosciences) pre-digested with the same restriction enzymes. Lentiviral vectors expressing RAD51 and RAD52 under the EF1α promoter were constructed by subcloning of a NcoI(blunt)-BamHI fragment containing RAD51 from pFB530 (54) or a NdeI(blunt)-BamH1 fragment containing RAD52 from pFB581 (55) (kindly provided by Dr S.C. West, Cancer Research UK) into the lentiviral vectors pCDH-EF1α-MCS-IRES-Neo and pCDH-EF1α-MCS-IRES-Puro (System Biosciences) cut by EcoRI(blunt)-BamHI.

Lentiviral vectors expressing CtIP and MRE11 were constructed by cloning blunted NotI-MruI fragments from pICE-HA-CtIP or pICE-HA-MRE11 into pCDH-EF1α-MCS-IRES-Neo cut with EcoRI and BamHI and blunted. All constructs were verified by sequencing.

Plasmid pCMV3-C expressing XAB2-GFPSpark was obtained from Sino Biological. The fusion construct was subcloned into pCDH-EF1α-MCS-IRES-Puro for long-term expression.

### CRISPR–Cas9 mClover–LMNA–HDR assay

U2OS cells were seeded in six-well plates. Knockdown by siRNA at 50 nM was performed 6 h later using Lipofectamine RNAiMAX (Invitrogen). Sixteen hours post-transfection, 10^6^ cells were pelleted for each condition and resuspended in 100 μl complete nucleofector solution (SE Cell Line 4D-Nucleofector X Kit, Lonza) to which 1 μg of pX330-LMNAgRNA, 1 μg pCR2.1-mClover-LMNAdonor and 0.1 μg of 2xNLSiRFP670 were added. Once transferred to a 100 μl Lonza certified cuvettes, cells were transfected with the 4D-Nucleofector X-unit using the program CM-104 and transferred to a 10 cm dish. After 30 h, cells were trypsinized and plated onto glass coverslips. Expression of the mClover in transfected cells (2xNLSiRFP670 positive population, *n* > 500 cells) was assayed the next day by fluorescence microscopy using the Zeiss CellDiscoverer 7.

### DNA fiber assay

Control and XAB2-depleted U87 cells were sequentially labelled first with 20 μM 5-iodo-2′-deoxyuridine (IdU) for 30 min and then with 100 μM 5-chloro-2′-deoxyuridine (CldU) for 30 min or with 100 μM CldU and 2.5 μM CPT for 60 min. Two thousand cells were loaded onto a glass slide (StarFrost) and lysed with spreading buffer (200 mM Tris–HCl pH 7.5, 50 mM EDTA, 0.5% SDS) by gently stirring with a pipette tip. The slides were tilted slightly and the surface tension of the drops was disrupted with a pipette tip. The drops were allowed to run down the slides slowly, then air dried, fixed in methanol/acetic acid 3:1 for 10 min, and allowed to dry. Glass slides were processed for immunostaining with mouse anti-BrdU to detect IdU, rat anti-BrdU to detect CldU, mouse anti-ssDNA antibodies (mouse anti-BrdU clone B44 (Becton Dickinson, 1/100), rat anti-BrdU clone BU1/75 (AbCys SA, 1/100) and ssDNA (HSHB auto anti-ssDNA, 1/100)), and corresponding secondary antibodies conjugated to various Alexa Fluor dyes. Nascent DNA fibers were visualized using immunofluorescence microscopy (Zeiss AxioImager Z2 ApoTome) at 40× magnification. The acquired DNA fibre images were analyzed by using MetaMorph Microscopy Automation and Image Analysis Software (Molecular Devices) and statistical analysis was performed with GraphPad Prism (GraphPad Software). The length of at least 100 CldU tracks were measured per sample.

### Cell cycle analysis by flow cytometry

Cells (2 × 10^5^) were harvested, fixed in 70% ethanol and stored overnight at -20°C. Thirty minutes before analysis, the samples were washed with PBS, treated with 0.2 mg/ml RNase A and stained with 20 μg/ml propidium iodide in PBS containing 0.1% Triton X-100. Cell cycle distribution was analyzed using a BD FACS Canto II flow cytometer.

### Immunofluorescence (IF) analysis

Cells grown on glass coverslips were incubated with TMZ (or DMSO) for 2 h at 37°C, washed once with media and left to recover in drug-free media for the indicated periods. Cells were then fixed with 4% paraformaldehyde in PBS for 10 min at room temperature (RT), permeabilized for 10 min with 0.5% triton X-100 in PBS and incubated for 30 min at RT in PBS with 2% bovine serum albumin (BSA) to block nonspecific binding. Thereafter, the cells were incubated with primary antibodies at 4°C (90 min or overnight, depending on the antibody), washed 3 times with PBS and then incubated with secondary antibodies for 1 h at RT. Cells were counterstained with DAPI and visualized using a Zeiss LSM880 confocal microscope.

For the visualization of Ku foci, cells exposed to DNA damaging agents or vehicle were subjected to pre-extraction using cytoskeleton (CSK) buffer containing 0.7% Triton X-100 and 0.3 mg/ml RNase A (CSK + R) as described (56). Cells were then fixed with 4% paraformaldehyde in PBS for 15 min at RT, blocked in PBS containing 10% fetal bovine serum and 1% BSA and then incubated with the primary antibodies for 90 min at RT in PBS containing 10% fetal bovine serum and 1% BSA. Following three washes with PBS, cells were incubated with the secondary antibodies for 60 min at RT in PBS containing 10% fetal bovine serum and 1% BSA, followed by washes and DAPI counterstaining. The ATMi KU-55933 (Tocris Biosciences) was used as described in experiments with CPT (56) and a similar protocol was adopted for the ATRi AZD6738 (Selleckchem). KU-55933 was added during the last half of the 48 h recovery period following exposure to TMZ.

Analysis of DNA end resection using immunofluorescence visualization BrdU-labelled ssDNA was carried out as previously described (57) with small modifications. In brief, cells were pre-incubated in the presence of 10 μM BrdU (Sigma-Aldrich) for 24 hours before the treatment with 1 μM CPT for one hour followed by a release of one hour. Cells were subjected to *in situ* fractionation on ice for 10 min using sequential extraction with two different buffers. Pre-extraction buffer 1 (10 mM PIPES, pH 7.0, 300 mM sucrose, 100 mM NaCl, 3 mM MgCl_2_ ,1mM EGTA and 0.5% Triton-X100) and followed by pre-extraction buffer 2 (10 mM Tris pH 7.5, 10 mM NaCl, 3 mM MgCl_2,_ 1% Tween20 and 0.5% sodium deoxycholate). Cells were washed three times with PBS followed by fixation with 4% paraformaldehyde (w/v) for 15 min at room temperature. Cells were then fixed for 5 min with methanol at -20°C. Cells were washed with PBS, permeabilized in 0.5% Triton X-100 in PBS for 10 min and incubated with blocking buffer (PBS + 5% BSA) for one hour. Cells were incubated overnight at 4°C with anti-BrdU and anti-PCNA antibodies in blocking buffer. Unbound primary antibody was removed by washing in PBS at room temperature followed by incubation with the secondary antibodies in PBS + 1% BSA for 1 h at room temperature followed by washes and DAPI counterstaining. Coverslips were mounted onto slides with ProLong Gold antifade mountant. BrdU foci were visualized on a DMI6000B microscope.

### Metaphase spread preparation and analysis

U87 cells (500 000 cells) exposed to TMZ or vehicle as before were allowed to recover for 45 h before being incubated in the presence of 0.5 μg/ml colcemid for a further 3 h at 37°C. Following trypsination and harvesting, cells were then swelled in 5 ml of pre-warmed hypotonic solution (0.54% KCL) added dropwise and incubated at 37°C for 30 min. Thereafter, 5 ml of freshly made, ice-cold fixing solution (ethanol:acetic acid (3:1, v/v)) were added and the preparations were stored overnight at 4°C. Metaphase preparations were dropped onto wet slides, air dried and stained with DAPI for microscopic visualization.

### Neutral Comet assay

Cell were embedded in 0.6% low melting agarose (LMA) and layered onto 0.6% normal agarose pre-coated frosted slides. Slides were then immersed in pre-chilled lysis buffer (2.5 M NaCl, 10 mM Tris–HCl, 100 mM EDTA pH8.0, 0.5% triton X-100, 3% DMSO, pH 9.5) for 1.5 h and then washed twice with pre-chilled distilled water (2 × 10 min) and twice with pre-chilled electrophoresis buffer (300 mM sodium acetate, 100 mM Tris–HCl, 1% DMSO, pH8.3) (2 × 10 min). Slides were equilibrated in fresh electrophoresis buffer for 1 h and then subjected to electrophoresis at 25 V (0.6 V/cm) for 1 h at 4°C. DNA was stained with DAPI, imaged with Zeiss LSM 510 confocal laser scanning microscope and pictures were quantified using Perceptive Instruments Comet Assay IV.

### Protein extracts and western blot analysis

Cells were harvested, washed once with ice-cold PBS and incubated in 1× RIPA buffer (Millipore, 20-188) supplemented with protease inhibitor cocktail (Roche, 11697498001) and phosphatase inhibitor cocktail (Roche, 04906837001) for 15 min at 4°C. Following centrifugation (16 000 g, 15 min) at 4°C, the lysates were stored at –20°C.

Protein extracts were quantified using the BRADFORD-solution (Bio-RAD, 5000006) and heated for 5–10 min at 95°C in LDS-sample loading buffer (Life Technologies, NP0008) containing 50 mM dithiothreitol (Amersham Biosciences, ref. 17-1318-02) before being subjected to SDS-PAGE gel electrophoresis (NuPage™ 4–12% Bis–Tris Gel Invitrogen, NP0322box) and semi-dry transfer to a nitrocellulose membrane (iBlot^®^2NC Mini Stacks Invitrogen, IB23002). The membrane was blocked in PBS containing 0.05% tween-20 (PBST) and 5% dry milk for 1 h at RT and incubated overnight with the appropriate primary antibody. Following three washes over 10 min with PBST the membrane was then incubated with the appropriate horseradish peroxidase (HRP)-conjugated secondary antibody in PBST containing 5% dry milk for 1–2 h at RT. Following three washes over 10 min with PBST at RT, signals were detected using ImageQuant LAS400 (General Electric) and images were captured using a SuperSignal™ West Pico Plus Chemiluminescent Substrate (Thermo Scientific) or SuperSignal™ West Femto Maximum Sensitivity Substrate (Thermo Scientific).

### Protein extracts and co-immunoprecipitations

U2OS cells (in 15-cm plates) were treated with DMSO or 1 μM CTP for 1 h and collected by scraping in cold PBS and centrifugation. Cells lysates were prepared by resuspending the cell pellets in IP lysis buffer (20 mM Tris–HCl pH 7.8, 150 mM NaCl, 1 mM EDTA, 0.5% NP-40) supplemented or not with 0.2 mg/ml RNase A and incubation for 30 min at 4°C, with vortexing (10 s) every 10 min. Extracts were collected following centrifugation at 15 000 rpm for 20 min. For co-IPs, extracts (500 μg of proteins) were brought to 300 μl with lysis buffer and 500 μl of IP dilution buffer (20 mM Tris–HCl pH 7.8, 150 mM NaCl, 1 mM EDTA, 0.05% NP-40) were added. Fifty microliter of Dynabeads Protein G (Life Technology) coated with a rabbit anti-XAB2 antibody (abcam, ab129487) or rabbit IgGs (Jackson ImmunoResearch Laboratories) were added and the mixture was incubated for 4 h on a spinning wheel at 4°C. The beads were then washed three times with IP wash buffer (20 mM Tris–HCl pH 7.8, 500 mM NaCl, 1 mM EDTA and 0.05% NP-40) and eluted in 50 mM glycine pH 2.8. The eluted samples were mixed with NuPAGE LSD sample buffer and heated for 10 min at 70°C, followed by separation on a NuPAGE 4–12% Bis–Tris gel (Invitrogen) and immunoblotting.

### Colony formation assays

Colony formation assays using U87 cells were carried out as follows: 300 cells were seeded onto 10 cm dishes and treated with TMZ for 2 h at 37°C in a humidified atmosphere of 5% CO_2_. The medium was then replaced and cells were allowed to form colonies for 15 days. Colonies were stained using crystal violet solution (0.1% Brilliant blue R (Sigma-Aldrich) in PBS) and quantified using ImageJ/FIJI analysis software.

For NCH644 cells, soft agar colony formation assays were carried out as follows: 6 × 10^3^ NCH644 cells were suspended in 0.3% low melting agarose (LMA) containing 1 ml Neural Stem Cell media (NSC-media, Gibco) supplemented with TMZ or vehicle, and then seeded on top of pre-coated 0.6% LMA (1 ml) containing NSC-media in the six-well plate. Cells were incubated for 21 days at 37°C with twice-weekly fresh medium supplementation (200 μl). After 3 weeks the cells plates were stained with crystal violet for imaging with ImageQuant TL and colony quantification.

### Cell proliferation assays

NCH644 cells were seeded in medium containing TMZ or vehicle (0.6 × 10^6^ cells in 5 ml). They were then split every 3–4 days under subconfluent conditions while remaining under TMZ or DMSO treatment. At each time point, the cumulative population doublings (PD) was calculated as follows:

$$\begin{equation*} {\rm{PD}}( {{t}} ) = {{\rm PD}_{( {t - 1} )}} + \frac{\log \left(\frac{{}{N_{(t)}}}{N_{(t-1)}}\right)}{\log(2)} \end{equation*}$$

where *N*(*t*) is the number of cells counted at time (*t*) and *N*(*t* –1) is the number of cells seeded at the previous time point, (*t* – 1). Cumulative population doublings were plotted against time.

Real-time cell proliferation analyses with U87 cells were carried using a xCELLigence Real-Time Cell Analyis (RTCA) instrument (ACEA Biosciences Inc.) in a 96-well format.

### Primary and secondary antibodies used for IF

Primary antibodies: XAB2 (abcam, ab129487, dilution 1:1000), RAD51 (Merck, Ab-1, PC130, dilution 1:1000), γH2AX (Millipore, Cat. No. 05-636, Clone JBW301, 1:1000), 53BP1 (abcam, ab36823, 1:1000), pS1778-53BP1 (Cell signalling, 26755, 1:1000), pS25/29-53BP1 (Cell signalling, 2674S, 1:1000), pRPA32 (S4/S8) (Bethyl Laboratories, A300-245A, 1:1000), Ku80 (abcam, ab80592, 1:1000), BrdU (GE Healthcare, RPN202, dilution 1:1000), PCNA (Chromotek, 16D10-100, 1:500). Secondary antibodies: Alexa Flour 647 (Invitrogen, A-21235, 1:500), Alexa Flour 555 (Invitrogen, A-21422, 1:500), Alexa Flour 488 (Invitrogen, A-11001, 1:500), Alexa Fluor 568 (Invitrogen, A-11077, 1:500).

### Primary and secondary antibodies used for western blotting

Primary antibodies: RAD51 (Merk, Ab-1, PC130, 1:1000), RAD52 (Thermo Fisher, PA5-65036, 1:500), XAB2 (abcam, ab129487, 1:1000), CtIP (Active Motif, 61141, 1:500), ATM (abcam, ab32420 (Y170), 1:1000), P-ATM (abcam, ab81292 (pS1981), 1:50000), P-KAP1 (Bethyl, IHC-00073 (pS824) 1:200).

Secondary antibodies: HRP rabbit (Jackson Laboratory, Cat. No. 111-035-003, 1:50 000), HRP mouse (Amersham/Sigma, Cat. No. GENA931-1ML, 1:10 000), HRP mouse (Santa Cruz, sc-516102, 1:10 000).

### Statistics and reproducibility

All the error bars are the standard error of mean (s.e.m.), unless mentioned otherwise in the legend. All the graphs are derived from two to five independent repeats. According to the number of samples, statistical significance (*P* values) of the difference between the means was determined by student *t-*test (two-tailed, upaired), one- or two-ways ANOVA. In the case of non-normally distributed datasets, non-parametric statistical tests have been preferred: Mann–Whitney test for comparisons between two samples and Kruskal–Wallis test for multiple comparisons. Statistical significance was always denoted as follow: ns = not significant; *P < 0.05; **P < 0.01; ***P < 0.001; ****P < 0.0001. All statistical analysis was performed using GraphPad Prism 8 (GraphPad Software).

## RESULTS

### XAB2 is important for the repair of seDSBs associated with O^6^-meG lesions left unrepaired by MGMT

**Figure 1.**
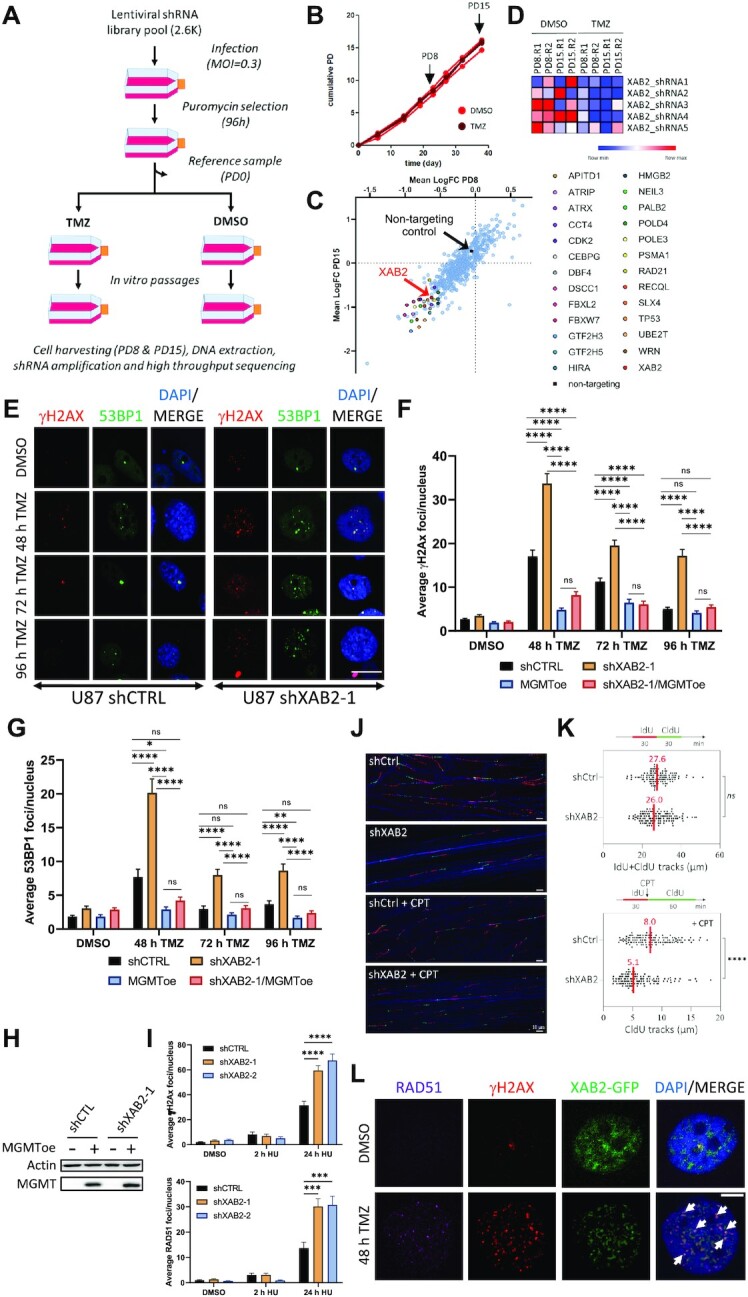
XAB2 depletion impairs the repair of DSBs associated with unrepaired O6-meG lesions induced by temozolomide. (**A**) Outline of the screen. NCH644 cells transduced with a lentiviral shRNA pool targeting 574 DDR genes were split into two arms exposed to TMZ or vehicle (DMSO), respectively. DNA libraries prepared from DNA extracted after 8 and 15 cumulative population doublings (PD) were sequenced using a MiSeq platform. MOI: multiplicity of infection. (**B**) NCH644 cell proliferation assessed under TMZ or DMSO during the screen replicates, showing the PD8/PD15 cell harvesting time points. (**C**) Means of the shRNA fold changes (FC) (TMZ versus DMSO) for each gene in the library at PD15 versus PD8. Prioritized hits are highlighted. (**D**) Heatmap showing the relative depletion of the five *XAB2*-targeting shRNAs at the indicated PD in both screen replicates (R1, R2). Normalized counts are depicted using a red (high) to blue (low) color key. (**E**) Representative immunofluorescence images used for γH2AX (red) and 53BP1 (green) foci quantification in control and XAB2-depleted U87 cells following exposure to 15 μM TMZ (or DMSO) and recovery for the indicated periods (scale bar = 5 μm). (**F** and **G**) Average γH2AX foci (**F**) and 53BP1 foci (**G**) per nucleus in control and XAB2-depleted cells as well as derivatives overexpressing MGMT. (**H**) Immunoblot analysis of MGMT overexpression. (**I**) Loss of XAB2 leads to increased accumulation of seDSBs associated with collapsed replication forks. U87 cells expressing the indicated shRNAs were incubated with hydroxyurea (HU) for 2 or 24 h, followed by immunofluorescence analysis and quantification of γH2AX (upper panel) and RAD51 (lower panel) foci. (**J** and **K**) DNA fiber assay. Control and U87-depleted cells were labeled with IdU and then CldU in the presence or absence of CPT. Replication fork progression was measured using the DNA fiber assay. Representative DNA spreads (**J**) and quantification of the length of IdU + CldU tracks in untreated cells or CldU tracks in CPT-treated cells (**K**). Median lengths are indicated in red. At least 100 fibers of each sample were measured (*n* = 2). *P*-values were calculated with the two-sided Mann–Whitney rank-sum test. (**L**) XAB2-GFPSpark-expressing U87 cells were exposed to TMZ for 2 h and allowed to recover for 48 h before IF analysis for RAD51 (magenta) and γH2AX (red). White arrows highlight examples of colocalization between XAB2-GFPSpark, γH2AX and RAD51 foci (scale bar: 5 μm). Data are representative of ≥3 independent biological repeats. Bars represent mean ± s.e.m. Significant differences between specified comparisons were assessed by two-tailed *t* test (**F** and **G**) or Kruskal–Wallis test (**I**) (*P< 0.05; **P< 0.01; ***P< 0.001; ****P< 0.0001).

To identify novel TMZ sensitizers in GBM cells, we performed a pooled shRNA screen targeting 574 DDR genes for gene depletions that conferred long-term loss of proliferation in the presence of TMZ (but not vehicle) to the GBM cell line NCH644 (Figure 1A). The screen was carried out in duplicate, using TMZ at a clinically-achievable concentration of 60 μM under serum-free conditions and in a monolayer format to ensure uniform TMZ distribution. Cells were harvested following 8 and 15 cumulative population doublings for DNA extraction, library preparation and shRNA read counting via high-throughput sequencing (Figure 1B). Twenty-six genes were identified as prioritized candidates by overlapping the top-ranking genes from three analysis algorithms, including *XAB2* (Figure 1C and D). Although XAB2 was initially identified as a *Xeroderma pigmentosum* complementation group A (XPA)-interacting factor involved in nucleotide excision repair, transcription and pre-mRNA splicing (58,59), more recently it was shown to also participate in DNA repair by HR (32,33). As XAB2 contribution to TMZ-induced DDR was hitherto unexplored, we undertook to characterize it. We validated *XAB2* as a novel TMZ sensitizer using clonogenic assays with NCH644 cells (GBM cancer stem-like cell line) (Supplementary Figure S1A–C) and U87 cells (GBM adherent cell line) (Supplementary Figure S1D–F) expressing a control, non-silencing shRNA (shCTRL) or two independent shRNAs targeting XAB2.

Given the proposed role of XAB2 in promoting HR (33), we examined the impact of XAB2 depletion on the repair of DSBs induced by TMZ. We treated control and XAB2-depleted NCH644 cells with TMZ for 2 h and visualized γH2AX foci, a DSB marker, by indirect immunofluorescence (IF) after a 72 h recovery period in drug-free medium, corresponding to ∼2 cell cycles after DNA damage induction. Compared to control cells, XAB2 depletion led to a ∼2-fold increase in the number of foci observed following exposure to TMZ (Supplementary Figure S1G and H), and this accumulation was corroborated by comet assay analysis under neutral conditions, which monitors DSB formation (Supplementary Figure S1I and J).

To obtain more insights into the DSB repair defects associated with XAB2 depletion, we characterized adherent U87 GBM cells which are more amenable to IF microscopy. As observed with NCH644 cells, TMZ exposure led to an increase in γH2AX foci in control U87-shCTRL cells, displaying maximal foci accumulation at 48 h (i.e. about 2 cell cycles) and a return to background levels at the later time points (Figure 1E and F). TMZ exposure led to a stronger increase in γH2AX foci in XAB2-depleted cells compared to control cells. In addition, unlike for control cells, we observed only a moderate decrease in γH2AX foci at the later time points in XAB2-depleted cells (Figure 1E and F). Similar observations were made with another DSB marker, 53BP1 (Figure 1E and G). Thus, XAB2 is required for the repair of TMZ-induced DSBs.

To verify that the TMZ-induced DSBs arose from *O*^6^-meG lesions left unrepaired by MGMT, and since U87 cells do not express MGMT, we examined the impact of XAB2 depletion on TMZ-induced γH2AX and 53BP1 foci formation in otherwise isogenic U87 derivatives ectopically expressing MGMT (Figure 1H). Stable expression of MGMT prevented the accumulation of γH2AX and 53BP1 foci associated with XAB2 depletion at all time points (Figure 1F and G). Similarly, ectopic MGMT overexpression prevented TMZ-induced γH2AX foci accumulation in XAB2-depleted NCH644 cells (MGMT-positive) (Supplementary Figure S1K and L). Taken together, these results indicate that XAB2 promotes the repair of *O*^6^-meG-associated seDSBs.

To gain support for the notion that XAB2 operates at seDSBs resulting from collapsed replication forks, we examined cells treated with hydroxyurea (HU), which depletes the dNTP pool and causes fork stalling (upon short exposure) or collapse (upon long exposure) (60). As expected, short incubation with HU did not cause significant γH2AX foci accumulation in control and XAB2-depleted cells whereas prolonged exposure led to a significant increase in γH2AX and RAD51 recombinase foci accumulation in XAB2-depleted cells compared to control cells (Figure 1I). We next examined the impact of XAB2 depletion on replication fork progression by analysing DNA replication tracks using the DNA fiber assay. We labelled newly synthesized DNA in control and XAB2-depleted U87 cells with 5-iodo-2′-deoxyuridine (IdU) and then with 5-chloro-2′-deoxyuridine (CldU) in the absence or presence of CPT. DNA fibers were spread on glass slides and the incorporation of halogenated thymidine analogues was detected by immunofluorescence using specific antibodies. As shown in Figure 1J and K, loss of XAB2 had little or no impact on replication fork progression in untreated cells. However, it resulted in a ∼36% reduction in the length of the CldU tracks following CPT treatment, revealing the importance of XAB2 for replication fork progression on damaged DNA. Together, these data demonstrate a crucial role for XAB2 in the repair of seDSBs induced by replication fork collapse.

As XAB2 is composed essentially of 15 tetratricopeptide repeat (TPR) domains (58) and since TPRs function as protein interaction modules and multiprotein complex scaffolds (61), we sought evidence that XAB2 operated at seDSBs. Using U87 expressing a XAB2-GFPSpark fusion, we observed a predominantly diffuse nuclear signal in untreated cells, with granular structures that may represent splicing speckles (62) (Figure 1L). However, TMZ treatment resulted in the accumulation of XAB2-GFPSpark foci in close coincidence to γH2AX and RAD51 foci (Figure 1L), suggesting a direct role for XAB2 in HR-mediated seDSB repair.

### XAB2-depletion leads to the increased engagement of non-homologous end-joining for the repair of seDSBs during S phase

**Figure 2.**
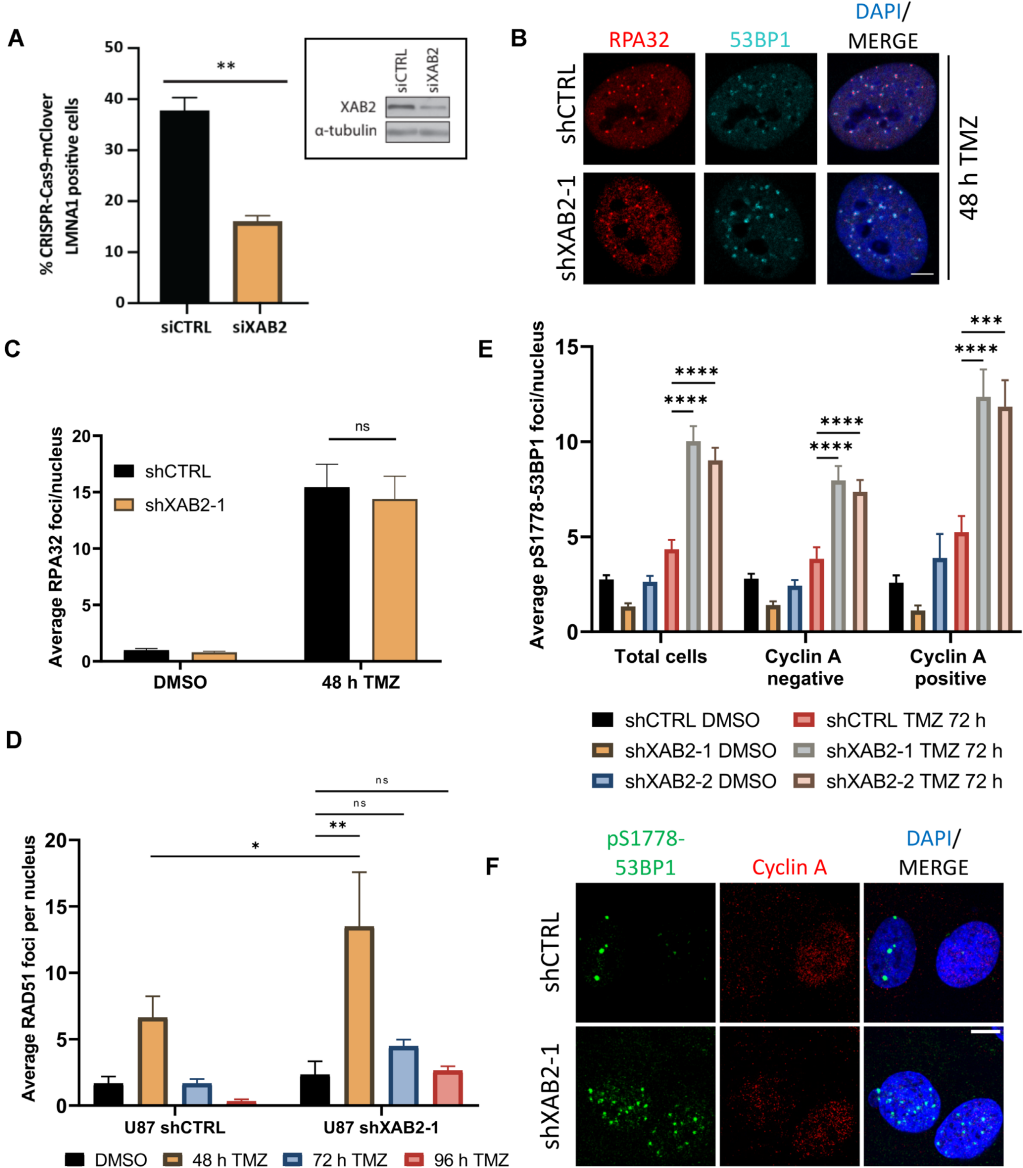
XAB2 depletion leads to the increased engagement of NHEJ in S/G2. (**A**) Quantification of the CRISPR–Cas9 mClover-LMNA1-HDR assay in control and XAB2-depleted U2OS cells. Data is representative of three independent biological repeats. Bars represent mean percentages of mClover-positive cells over the iRFP-positive population ± s.e.m. Significant differences between specified comparisons were assessed by unpaired t-test and are highlighted by stars (*P< 0.05; **P< 0.01; ***P< 0.001; ****P< 0.0001). Inset, efficiency of XAB2 depletion achieved by siRNA (siXAB2) as compared to a non-silencing control siRNA (siCTRL). α-Tubulin was used as a loading control. (**B** and **C**) Representative immunofluorescence images of RPA32 foci (red) and 53BP1 foci (green) in control and XAB2-depleted U87 cells exposed to 15 μM TMZ (or DMSO) for 2 h and allowed to recover in drug-free medium for 48 h (**B**), and related quantification of the average number of RPA32 foci per nucleus (**C**). (**D**) Quantification of the average number of RAD51 foci in control and XAB2-depleted cells exposed to 15 μM TMZ (or DMSO) for 2 h and allowed to recover in drug-free medium for the indicated times. See Supplementary Figure S2 for representative immunofluorescence images. (**E** and **F**) Quantification of the average number of pS1778-53BP1 foci per nucleus, in the total cell population, as well as in Cyclin A-negative and -positive cell subpopulations, in control and XAB2-depleted U87 cells following exposure to 15 μM TMZ (or DMSO) for 2 h, 72 h recovery in drug-free medium and processing for IF analysis of pS1778-53BP1 foci and Cyclin A (**E**), and representative immunofluorescence images (**F**). Scale bar: 5μm. The images are representative of three or more independent biological repeats. Bars represent mean ± s.e.m. Significant differences between specified comparisons were assessed by two-ways ANOVA and are highlighted by stars (*P< 0.05; **P< 0.01; ***P< 0.001; ****P< 0.0001).

As HR is the preferred repair pathway for seDSBs resulting from collapsed replication forks, we first examined the integrity of the HR machinery in XAB2-depleted cells using a CRISPR–Cas9 mClover-LMNA1-HDR assay (63). This assay measures the homology-directed repair (HDR)-dependent insertion of mRuby2 into a Cas9-mediated two-ended DSB in the *LMNA* gene, resulting in cellular expression of mRuby2-tagged lamin A*/*C (LMNA) that serves as readout for HDR activity. As shown in Figure 2A, siRNA-mediated depletion of XAB2 impaired HR significantly, in line with a previous study (33).

As RAD51 acts upon RPA-coated resected seDSBs, we next examined RPA and RAD51 foci formation in TMZ-treated cells. Control and XAB2-depleted cells displayed a similar increase in RPA foci 48 h after TMZ treatment, indicating efficient end resection (Figure 2B and C). Moreover, loss of XAB2 led to an increased accumulation of RAD51 foci under these conditions (Figure 2D and Supplementary Figure S2), which paralleled the increase in γH2AX foci already seen at this time point (Figure 1E and F). Notably, unlike γH2AX and 53BP1 foci, RAD51 foci did not accumulate significantly at the later time points (Figure 2D). As XAB2 depletion did not affect the percentage of cells in S/G2 and G1 phase at the considered time points as assessed by Cyclin-A staining (Supplementary Figure S3A), and did not impact cell cycle phase distribution as assessed by flow cytometry analysis (Supplementary Figure S3B and C), the persistence of γH2AX and 53BP1 foci observed at the later time points suggested that, in the absence of XAB2, RAD51 did not act properly on a subset of seDSBs induced by TMZ.

We next tested if NHEJ gained more prominence in XAB2-depleted cells by examining foci formation by the Ser1778-phosphorylated form of 53BP1 (pS1778-53BP1), a modification mediated by ATM (64,65) and involved in NHEJ (66,67) and the repair of broken replication forks (68). Loss of XAB2 significantly increased the number of TMZ-induced pS1778-53BP1 foci (Figure 2E and F). We confirmed this observation using an antibody against pS25/29-53BP1 (69,70) and verified the increased phosphorylation of 53BP1 at these sites by western blot analysis (Supplementary Figure S4A–C). To relate our findings to cell cycle stages and distinguish cells in S/G2 from G1 cells, we also visualized Cyclin A. In Cyclin A-negative (G1) cells, XAB2 depletion caused an increase in the number of TMZ-induced pS1778-53BP1 foci observed after 72 h (Figure 2E and F), consistent with the extra burden of unrepaired damage associated with defective HR. Importantly, under these conditions, XAB2 loss also resulted in a ∼2.4-fold increase in pS1778-53BP1 foci in Cyclin A-positive (S/G2) cells (Figure 2E and F). Furthermore, the percentage of S/G2 cells with more than five pS1778-53BP1 foci reached ∼87% in TMZ-treated XAB2-depleted cells, compared to ∼41% in control cells (Supplementary Figure S4D). A comparable increase in NHEJ engagement in S/G2 was already observed at 48 h (Supplementary Figure S4D).

One prediction of the increased engagement of NHEJ in the repair of TMZ-induced seDSBs in XAB2-depleted S/G2 cells is that it should be associated with increased genetic instability. Consistent with this notion, loss of XAB2 increased the number of chromosomal aberrations detected in metaphase spreads by 2.5-fold following TMZ exposure (Supplementary Figure S4E and F).

### Loss of XAB2 is associated with Ku retention at resected DNA repair intermediates and abortive homologous recombination

**Figure 3.**
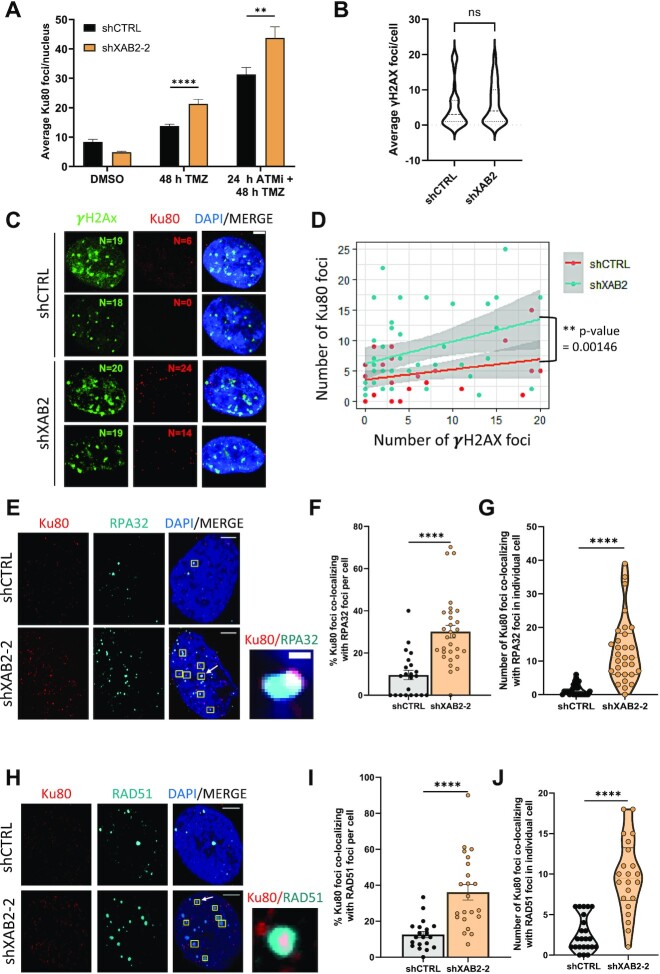
XAB2 prevents Ku retention at seDSBs induced by temozolomide. (**A**) Quantification of the average number of Ku80 foci detected by immunofluorescence microscopy in control and XAB2-depleted U87 cells exposed to 15 μM TMZ (or DMSO) for 2 h and allowed to recover for 48 h in the absence or presence of the ATMi KU-55933. (**B–D**) Linear regression analysis of the correlations between Ku80 foci formation, γH2AX foci formation and XAB2 depletion. Control and XAB2-depleted U87 cells were treated with TMZ for 2 h and allowed to recover in TMZ-free medium for 48 h before being processed for IF visualization of Ku80 and γH2AX. To enable comparison between control and XAB2-depleted cells, only cells displaying ≤20 γH2AX foci (corresponding to the average number of foci observed in TMZ-treated, control cells examined after 48h (Figure 1F)) were considered. (**B**) Violin plot showing the distribution of γH2AX foci number among the cells with ≤20 γH2AX foci analyzed in (C, D). (**C**) Representative IF images illustrating the increased accumulation of Ku80 foci (red) in XAB2-depleted cells compared to control cells displaying similar numbers of γH2AX foci (green). Indicated are the numbers of γH2AX and Ku80 foci. (**D**) Scatter plot displaying the relationship between Ku80 and γH2AX foci in control and XAB2-depleted cells, used for linear regression analysis. The difference between the two regression lines was calculated using a linear model with two variables under the assumption that Ku80 foci formation is affected by both the number of γH2AX foci and XAB2 depletion. The statistical significance of the resulting coefficients was reported in the form of *P*-values. The grey areas represent the confidence interval of the linear model. (**E**) Representative immunofluorescence images used for the quantification of the number of colocalizing Ku80 (red)-RPA32 (cyan) foci following exposure of control and XAB2-depleted U87 cells to TMZ and recovery for 48 h. Examples of colocalized foci are highlighted by yellow squares, with one representative example (indicated by an arrow) shown in the close up section (scale bar: 0.1 μm). (**F** and **G**) Percentage of colocalized Ku80-RPA32 foci per cell following exposure to TMZ (**F**), quantified based on foci examination in individual cells, as presented in a violin plot (**G**). (**H**-**J**) Same as (E–G) for the analysis of Ku80 (red) and RAD51 (cyan) foci colocalization (scale bar: 5 μm). The images are representative of three independent biological repeats. Bars represent mean ± s.e.m. Significant differences between specified comparisons were assessed by a *t*-test (unpaired, two-tails) and are highlighted by stars (*P< 0.05; **P< 0.01; ***P< 0.001; ****P< 0.0001).

Although our data indicate that resection and ssDNA/RAD51 associations can occur in the absence of XAB2, the increased engagement of NHEJ observed in S phase in XAB2-depleted cells suggests that loss of XAB2 inhibits HR in some way. Based on previous work (11), we considered the possibility that XAB2 depletion led to Ku persistence at seDSB termini. Following pre-extraction with Triton X-100 and RNase A (CSK + R) to enable IF analysis of damage-specific Ku retention on chromatin (56), we found that TMZ treatment led to a ∼1.5-fold increase in Ku80 foci in XAB2-depleted cells compared to control cells (Figure 3A). We verified that such an increase did not simply reflect the accumulation of γH2AX foci induced by XAB2 depletion by examining Ku80 foci in control and XAB2-depleted cells displaying comparable numbers of γH2AX foci (Figure 3B and C). We represented the relationship between the number of γH2AX and Ku80 foci in a scatter plot and analyzed the effect of both γH2AX foci number and XAB2 depletion on Ku80 foci using a linear regression model with two variables. As expected, there was a significant correlation between the number of γH2AX foci and Ku80 foci in both control and XAB2-depleted cells, based on the slope of the linear regression model on individual data (slope significantly different from zero at **P-value = 0.00646, Figure 3D). In addition, loss of XAB2 led to a significant increase in Ku80 foci irrespective of the increase in γH2AX foci, as showed by the difference between the intercepts of the shXAB2 and shCTRL regression lines (**P-value = 0.00146, Figure 3D), consistent with the notion that XAB2 prevents Ku accumulation at seDSB termini. ATM defines one pathway for Ku release at seDSBs induced by CPT (11,71). In agreement with these studies, inhibition of ATM kinase activity using the ATM inhibitor (ATMi) KU-55933 (72) also resulted in increased Ku80 foci formation in TMZ-treated cells (Figure 3A). Notably, ATM inhibition further increased the number of Ku80 foci elicited by XAB2 depletion (Figure 3A). Importantly, Ku80 foci detected following exposure to TMZ were associated with resected seDSBs as assessed by the accumulation of RPA foci. Thus, loss of XAB2 led to a ∼3-fold increase in the frequency of Ku80-RPA32 colocalized foci (Figure 3E–G) compared to control cells. Importantly, XAB2 depletion resulted in a ∼2.9-fold increase in the frequency of Ku80-RAD51 foci (Figure 3H–J). Of note, the colocalization data of Figure 3F and I suggest that XAB2 is required to prevent Ku retention at about a third of seDSB termini.

As the collapse of replication forks leading to seDSBs elicited by unrepaired O6-meG lesions follows a complex sequence of events involving successive rounds of DNA replication and cell division, we next sought to extend our findings to seDSBs induced in a more rapid manner by CPT, which can be monitored shortly after exposure to the topoisomerase I poison. Exposure to 1 μM CPT for 1 h generated equal levels of DNA damage in control and XAB2-depleted cells, as assessed by γH2AX foci formation (Supplementary Figure S5A). As seen for TMZ, CPT treatment led to an increase in Ku80 foci in XAB2-depleted cells compared to control cells (Supplementary Figure S5B and C), which was exacerbated in the presence of ATMi (Supplementary Figure S5B and C), in agreement with previous work (11). Furthermore, loss of XAB2 increased the frequency of CPT-induced Ku80-RPA32 colocalized foci (>2.5-fold) and Ku80-RAD51 colocalized foci (>1.7-fold) (Supplementary Figure S5D–F). Taken together, our data suggest that ATM and XAB2 identify separate pathways for Ku eviction from seDSB termini.

**Figure 4.**
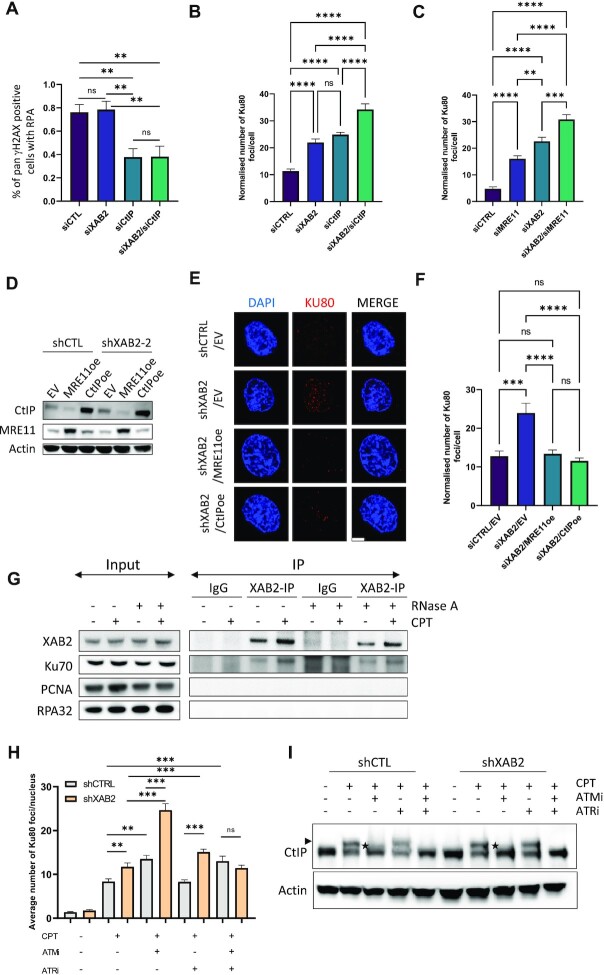
XAB2 operates in parallel to the ATM-CtIP-MRE11 axis for Ku eviction at seDSBs and it interacts with Ku. (**A**–**C**) The combination of XAB2 siRNAs with CtIP or MRE11 siRNAs has an additive effect on Ku foci accumulation in U2OS. (**A**) Percentage of γH2AX-positive cells with visible RPA foci in control cells as well as cells harbouring single or combined depletion of XAB2 and CtIP. (**B** and **C**) Quantification of Ku foci in cells transfected with XAB2 and/or CtIP siRNAs (**B**) or XAB2 and/or MRE11 siRNAs (**C**) 48 h before treatment with 1 μM CPT (or DMSO) for 1h and processing for Ku foci detection by IF. Data were normalized by subtraction of the foci observed in the DMSO condition. See Supplementary Figure S8A–D for immunoblotting analysis of XAB2, CtIP and MRE11 depletion, as well as representative IF images. (**D**–**F**) Overexpression of CtIP or MRE11 rescues the Ku foci accumulation phenotype of defective XAB2 cells. (**D**) Immunoblotting analysis of CtIP and MRE11 overexpression in control and XAB2-depleted U87 cells. (**E** and **F**) Representative micrographs of Ku foci (red) detected by IF in the indicated cells exposed to 1 μM CPT (or DMSO) for 2h and allowed to recover in drug-free medium for 48 h (**E**) and related quantification in (**F**) (scale bar: 5 μm). Bars represent mean ± s.e.m. Significant differences between specified comparisons were assessed by one-way ANOVA and are highlighted by stars (*P< 0.05; **P< 0.01; ***P< 0.001). (**G**) XAB2 interacts with Ku. Western blot analysis of Ku70, PCNA, RPA32 and XAB2 in immunoprecipitates obtained from untreated or CPT-treated (1 μM) U2OS cells using a non-specific IgG or an anti-XAB2 antibody. Presence of RNase A in the immunoprecipitation reactions is indicated. (**H** and **I**) Impact of ATM and ATR inhibition on Ku eviction from seDSBs and seDSB-induced CtIP phosphorylation in control and XAB2-depleted cells. (**H**) Quantification of the average number of Ku80 foci detected by immunofluorescence microscopy in control and XAB2-depleted U87 cells exposed to 1 μM CPT (or DMSO) for 1 h in the absence or presence of the ATMi KU-55933 and/or the ATRi AZD6738 (*n* = 3). Bars represent mean ± s.e.m. Significant differences between specified comparisons were assessed by a t-test (unpaired, two-tails) and are highlighted by stars (*P< 0.05; **P< 0.01; ***P< 0.001). (**I**) Immunoblot analysis of CtIP in cell extracts from control and XAB2-depleted U87 cells exposed to 1 μM CPT for 1 h in the absence or presence of ATM and/or ATR inhibitors. The arrowhead and asterisks indicate shifts in CtIP electrophoretic mobility observed following CPT induction alone or in the presence of ATMi, respectively.

**Figure 5.**
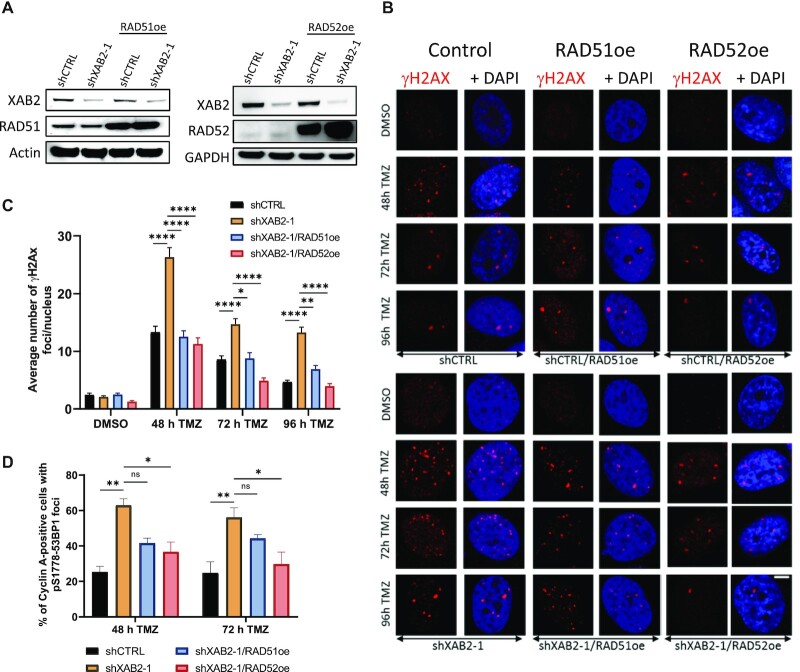
Rescue of defective XAB2 by overexpression of RAD51 and RAD52. (**A**) Immunoblotting analysis of RAD52 (upper panel) and RAD51 (lower panel) overexpression in control and XAB2-depleted U87 cells. (**B** and **C**) Representative micrographs of γH2AX foci (red) detected by immunofluorescence in the indicated cells exposed to 15 μM TMZ (or DMSO) for 2 h and allowed to recover in drug-free medium for the indicated times (**B**) and related quantification (**C**) (scale bar: 5 μm). (**D**) Percentage of Cyclin A-positive cells displaying pS1778-53BP1 foci among control and XAB2-depleted cells harboring the indicated constructs, following exposure to 15 μM TMZ (or DMSO) for 2 h and recovery in drug-free medium for the indicated times. Data are the average of *n* = 2 or more biological replicates (30–50 cells/sample/experiment). The images are representative of three independent biological repeats. Bars represent mean ± s.e.m. Significant differences between specified comparisons were assessed by two-ways ANOVA and are highlighted by stars (*P< 0.05; **P< 0.01; ***P< 0.001; ****P< 0.0001) (ns = not significant).

In a previous study investigating the role of XAB2 in HR in the U2OS human osteosarcoma cell line, siRNA-mediated depletion of XAB2 resulted in a decrease in CPT-induced chromatin-bound RPA, as assessed by flow cytometry analysis, which was interpreted as indicative of defective end resection (33). However, similar to U87 cells, we found that shRNA-mediated XAB2 depletion in U2OS cells did not prevent RPA32 and RAD51 foci formation following exposure to CPT (Supplementary Figure S6A–F), suggesting that end resection was not impaired in these cells. Ruling out possible effects linked to the shRNA approach used to deplete XAB2, similar results were obtained using specific siRNAs targeting XAB2 (Supplementary Figure S7A–C). To complement our analysis of RPA foci, we monitored DNA end resection in the siRNA-treated cells through IF visualization of bromodeoxyuridine (BrdU)-labelled ssDNA. XAB2 depletion did not decrease the number/intensity of BrdU foci elicited by CPT compared to control cells (Supplementary Figure S7D–G). Notably, XAB2 depletion did not affect the levels of CPT-induced hyper-phosphorylation of ATM (Supplementary Figure S7H). Finally, as Sakasai *et al.* have shown that the depletion of the splicing factor Aquarius elicited a decrease in the protein levels of CtIP in a cell-type specific manner while that of XAB2 led to decreased CtIP and RAD51 levels in HCT116 cells (32), we verified that the protein levels of CtIP, RAD51, Ku and RPA32 were not affected by XAB2 loss in U2OS as well as in U87 and NCH644 cells (Supplementary Figure S7I; see also Figures 4I and 5A).

Phosphorylations of the RPA-coated ssDNA platform by ATM, ATR and DNA-PK play crucial roles in the DDR (73). Previous studies have identified the splicing factor PRP19, which is found together with XAB2 and Aquarius in various splicing complexes (74), as a sensor of the RPA-coated ssDNA platform promoting HR promotion at DNA damaged sites (75,76). Notably, while knockdown of PRP19, caused only a mild reduction in CPT-induced RPA32 foci, it resulted in a dramatic reduction in pS4/S8-RPA32 foci (75,76). Of note, XAB2 was not present in the list of proteins that associated with RPA-coated ssDNA in the proteomic analysis of Maréchal *et al.* (75). DNA-PK-mediated phosphorylation of RPA32 at Ser-4 and Ser-8 was identified as a mark of transient Ku association with seDSBs (11). In agreement with its associated Ku-retention phenotype, we found that XAB2 depletion elicited a small but significant increase in pS4/S8-RPA32 foci formation following exposure to CPT (Supplementary Figure S7J and K).

Collectively, our observations suggest that XAB2 is not required for seDSB end resection or modification of the RPA-coated ssDNA platform but prevents Ku retention and abortive HR at seDSBs induced by CPT and TMZ.

### XAB2 operates in parallel to the ATM-CtIP-MRE11 axis to prevent Ku retention at seDSBs, and interacts with Ku

Having shown that ATM inhibition and XAB2 depletion had an additive effect on the accumulation of CPT-induced Ku foci, we tested epistatic relationships between XAB2, CtIP and MRE11. As expected, siRNA-mediated CtIP depletion led to a severe decrease in DNA end resection in CPT-treated U2OS cells, which XAB2-depletion did not exacerbate (Figure 4A and Supplementary Figure S8A). Compared to the single depletion of XAB2, combined siRNA-mediated depletion of XAB2 and either CtIP or MRE11 in U2OS led to an additive effect on Ku foci accumulation at seDSBs induced by CPT (Figure 4B, C and Supplementary Figure S8A–D). Moreover, overexpression of CtIP or MRE11 rescued the accumulation of Ku foci induced by CPT in XAB2-depleted U87 cells (Figure 4D–F). Taken together, these observations indicate that XAB2 operates in parallel to the ATM-CtIP-MRE11 axis for prevention of Ku retention at seDSBs.

To gain more insights into the mechanisms whereby XAB2 promotes Ku removal, we next carried out co-immunoprecipitation (co-IP) assays to examine whether XAB2 physically interacted with Ku. We found that XAB2 interacted with Ku in untreated as well as CPT-treated cells (Figure 4G). The interaction was resistant to RNase A-treatment, suggesting that it was not mediated by RNA. Under the conditions used for Ku detection, PCNA was not detected in the XAB2-immunoprecipitates. Likewise, RPA32 was not present in the co-IPs. These data suggest that XAB2 does not travel with replication forks but is recruited by Ku to damaged DNA sites. They further suggest that XAB2-mediated Ku removal form seDSBs may occur through a direct mechanism involving their interaction.

Given that XAB2 operates in parallel to ATM for the removal of Ku from seDSBs, and prompted by the presence of ATRIP, partner of the ATR signalling kinase in DNA damage sensing (77,78), in our list of TMZ sensitizers (Figure 1C), we investigated the involvement of ATR in the mechanisms leading to Ku eviction. We analyzed the impact of the ATR inhibitor (ATRi), AZD6738 (79), alone or in combination with KU-55933, on Ku retention at seDSBs induced by CPT in control and XAB2-depleted U87 cells. Unlike ATMi, addition of ATRi did not increase the numbers of Ku induced by CPT is U87-shCTL cells (Figure 4H). Furthermore, the effect of combined ATM and ATR inhibition was similar to that of inhibiting ATM alone (Figure 4H). Together, these data suggest that ATR does not contribute to Ku release from seDSBs when XAB2 is present. In contrast, Ku foci accumulation increased significantly, although to a lesser extent in comparison with ATMi, upon addition of ATRi to XAB2-depleted cells (Figure 4H), indicating that in the absence of XAB2, ATR contributes independently of ATM to prevent Ku accumulation at seDSBs. Notably, combined inhibition of ATM and ATR in XAB2-depleted cells did not increase further the number of Ku foci seen in control cells under the same conditions (Figure 4H), suggesting that, when these pathways were disrupted, alternative DSB mechanisms capable to displace Ku could operate at seDSBs. These could possibly involve microhomology-mediated end-joining (MMEJ) activities, including microhomology-mediated BIR (MMBIR). Indeed, MMEJ operates during S/G2, and replication-associated seDSBs have been shown to be repaired by MMBIR in HR-deficient cells unable to utilize RAD51-dependent BIR (14,80). Furthermore, MMEJ involves PARP-1, which is able to compete with Ku for binding to DSB ends (81) and recruit crucial nucleases involved in DNA resection, including MRE11 (57,81).

As ATM-dependent phosphorylation of CtIP is crucial to potentiate its Ku eviction activity (11) and since ATM and ATR both phosphorylate several sites in CtIP (82–86), we studied the effect of inhibiting ATM and/or ATR on the hyperphosphorylation of CtIP induced by CPT in control and XAB2-depleted cells. In control cells, exposure to CPT led to CtIP species presenting a shift in electrophoretic mobility, indicative of its hyperphosphorylation (Figure 4I). ATM inhibition impaired CtIP hyperphosphorylation severely, but not completely, resulting in species of intermediate mobility (Figure 4I). Importantly, the combined inhibition of ATM and ATR abrogated CtIP hyperphosphorylation (Figure 4I). Notably, XAB2 depletion had no impact on the observed shifts in mobility (Figure 4I). Thus, like ATM (10,11), ATR appears to mediate some CtIP phosphorylations important to counter Ku accumulation at seDSBs. Such phosphorylations are not affected by XAB2 depletion.

### Rescue of the DNA repair deficiencies in XAB2-depleted cells by overexpression of RAD51 and RAD52

Previous studies in yeast have shown that RAD51 and RAD52 overexpression could suppress defective BIR, e.g. caused by dysfunctional RPA stabilization of ssDNA (87). We found that stable ectopic overexpression of RAD51 or RAD52 (Figure 5A–C) decreased the number of TMZ-induced γH2AX foci associated with XAB2 depletion to near background levels, with overexpressed RAD52 affording the strongest rescue at the later time points (Figure 5B and C). In line with this observation, analysis of the 72 h time points for Cyclin A and pS1778-53BP1 positivity, revealed that overexpression of RAD52, but not RAD51, reduced NHEJ engagement in S/G2 in XAB2-depleted cells to near background levels (Figure 5D).

### RAD52 inhibition is synthetically lethal with XAB2 depletion

**Figure 6.**
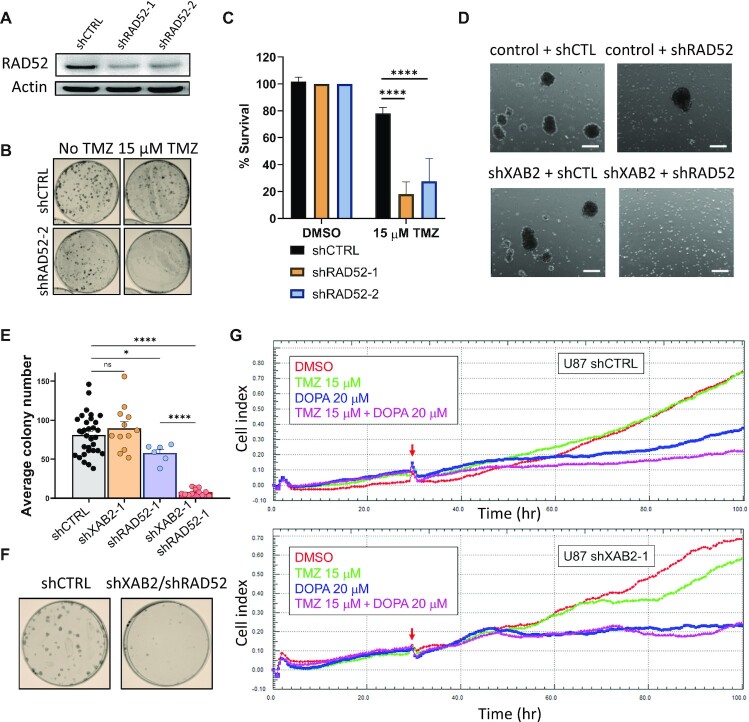
XAB2 depletion shows synthetic lethality with loss or inhibition of RAD52. (**A**) Immunoblots illustrating the efficiency of RAD52 depletion achieved by 2 independent shRNAs (shRAD52-1 and shRAD52-2) as compared to shCTRL. Actin was used as a loading control. (**B** and **C**) Clonogenic survival assays carried out with control and RAD52-depleted cells exposed to 15 μM TMZ (or DMSO) for 2 h (**B**) and related quantification (**C**). Cell viabilities are expressed as % relative to that of untreated cells (set at 100%). (**D**) Micrographs of control or XAB2-depleted NCH644 cells transduced with either shRAD52 or control (shCTL) shRNAs, taken at day 18 following transduction and selection in puromycin/G418 (scale bars: 250 μm). Control and XAB2-depleted cells, obtained using shRNA constructs expressing the puromycin-resistance marker, were transduced with pLKO-based constructs expressing shCTL or shRAD52-2 shRNAs, followed by additional selection with G418. No spheroid growth was observed in the double knockdown cells. (**E** and **F**) Quantification of the clonogenic survival of U87 cells following single or double knockdown of XAB2 and RAD52 (**E**) and representative illlustration of the plating efficiency of the double knockdown cells compared to control cells (**F**). See Supplementary Figures S1E and S5B for illustrations of the single knockdowns. (**G**) xCELLigence real-time cell proliferation analysis with control and XAB2-depleted cells exposed to the indicated concentrations of TMZ and/or L-DOPA, or vehicle. Red arrows indicate the time of drug addition. Images shown are the representative of three independent experiments. Data are the average of 3 or more biological replicates. Bars represent mean ± s.e.m. Significant differences between specified comparisons were assessed by one- or two-ways ANOVA and are highlighted by stars (*P< 0.05; ***P< 0.001).

Although we did not identify RAD52 in our shRNA screen, the significant rescue afforded by its overexpression in XAB2-depleted cells prompted us to explore its importance in the response of GBM cells to TMZ. shRNA-mediated RAD52 depletion in U87 cells led to a strong sensitivity to TMZ (Figure 6A–C). We next tried to generate cells harbouring the dual depletion of XAB2 and RAD52 using RNA interference. In NCH644, no viable double knockdown cells could be obtained (Figure 6D), suggesting synthetic lethality. In U87, we obtained double knockdown cells, but their clonogenic survival was severely impaired (Figure 6E and F).

To corroborate these observations, we carried out real-time cell proliferation analyses following RAD52 inhibition with 6-hydroxy-dl-DOPA (L-DOPA), which disrupts RAD52 ring structures and suppresses RAD52 recruitment and recombination activity in cells, with negligible effects on other DSB repair pathways (46), and was used in previous *in vitro* synthetic lethality experiments (88). In wild-type cells, TMZ had little or no impact on proliferation (Figure 6G), but exposure to L-DOPA alone impaired proliferation and its effect was exacerbated in combination with TMZ (Figure 6G, upper panel), consistent with the impaired clonogenicity of RAD52-depleted cells (Figure 6B and E). As expected, TMZ impaired the proliferation of XAB2-depleted cells. In addition, RAD52 inhibition by L-DOPA abolished the proliferation of XAB2-depleted cells even in the absence of TMZ (Figure 6G, lower panel).

## DISCUSSION

**Figure 7.**
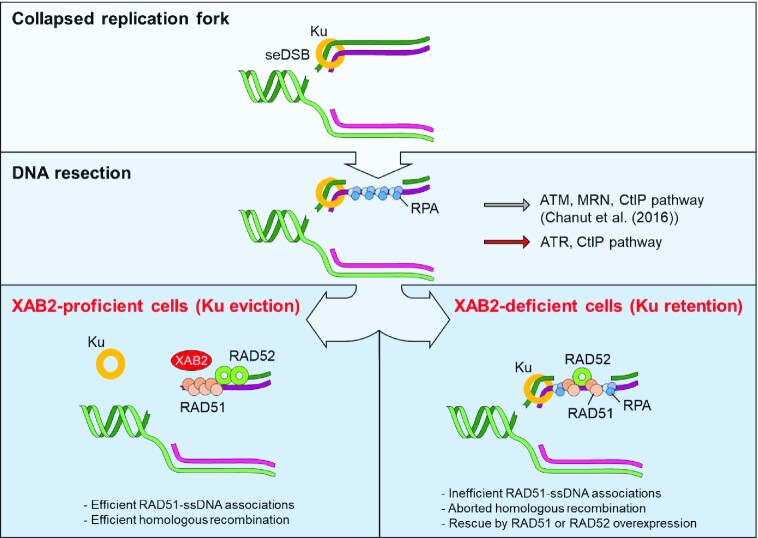
Pathways for Ku eviction—model integrating the present findings. XAB2 defines a novel pathway for Ku eviction from a subset of seDSBs resulting from collapsed replication forks. This pathway operates in parallel to an ATM/MRN/CtIP-dependent pathway previously described (11). Loss of XAB2 does not prevent interaction between RAD51 and ssDNA generated through end resection. However, the resulting associations are unproductive, leading to increased NHEJ engagement in S phase. Such defects can be rescued by overexpression of RAD51 or RAD52. In addition, ATR also contributes to CtIP phosphorylations and Ku removal at seDSBs. Our data suggest that ATR can operate in cooperation with ATM, as well as independently of ATM.

XAB2 was initially identified as a XPA-interacting factor involved in nucleotide excision repair, transcription and pre-mRNA splicing (58,59). In 2016, Onyango *et al.* (33) reported the first demonstration of a role for XAB2 in promoting HR. Since then, however, no further mechanistic insights have been provided. In this work, we show for the first time that XAB2 is required for efficient replication fork progression on damaged DNA and that it promotes the recombinational repair of seDSBs through a novel pathway promoting Ku eviction. Previously, an ATM kinase-dependent pathway involving CtIP and MRE11 was shown to promote Ku eviction from about 40% of seDSB termini, by a mechanism suggested to involve MRE11-CtIP cleavage-mediated release of a short DNA fragment containing Ku (11). Our data indicate that the XAB2 pathway operates in parallel to the ATM-CtIP-MRE11 axis (Figure 7). XAB2 has been shown to interact with several DNA repair factors (58,89). The physical interaction detected between XAB2 and Ku together with the observed recruitment of XAB2 in close proximity to γH2AX and RAD51 foci strongly suggest that XAB2 operates by a direct mechanism to promote Ku eviction at seDSBs. As XAB2 is composed essentially of TPR domains, we propose that XAB2 is recruited by Ku and serves as a scaffold for the assembly of processing and/or modifying factors that will mediate Ku removal from seDSBs. The molecular details of this novel pathway merit further exploration. Interestingly, our observation that overexpression of CtIP and MRE11 can rescue defective XAB2 suggests that the ATM–CtIP–MRE11 axis and the XAB2 pathway can compete for Ku eviction at seDSBs. The modalities of this competition remain to be determined.

Ku removal via the ATM-CtIP-MRE11 axis requires CtIP phosphorylation *in vivo* and *in vitro* (10,11,90). Our data support the notion that ATM plays a major role in promoting the CtIP phosphorylations that promote Ku eviction from seDSBs, consistent with previous studies (10,11). In addition, they reveal a new role for ATR, the apical DNA replication stress response kinase (5), in this process. Given the post-resection roles of ATR in HR (78,91) and CtIP phosphorylation (82), and consistent with the presence of ATRIP in our list of TMZ sensitizers, we propose that ATR cooperates with ATM, following recruitment and activation of the ATR-ATRIP complex by RPA-coated ssDNA. In addition, our data in XAB2-depleted cells suggest that ATR can operate independently of ATM to promote Ku eviction from seDSBs.

Defective MRE11 exonuclease compromises RAD51 focus formation at seDSBs (11). Our observations with XAB2-depleted cells suggest that, at least under certain instances, RPA-RAD51 exchange on ssDNA may not require coupling to Ku removal. However, overexpression of RAD51 rescued the DNA damage defect associated with XAB2 depletion, indicating that the ssDNA-RAD51 associations that take place on Ku-bound, resected seDSBs in the absence of XAB2 are not sufficient for efficient recombinational repair. In contrast, RAD52 can exploit microhomologies (92–94) and its transactions with DNA require moderate resection compared to RAD51-mediated filament formation. Furthermore, RAD52 competes with Ku for binding to DSB free ends generated in the switch region during antibody class-switch DNA recombination (93), suggesting the possibility that, unlike RAD51, it may be able to operate at seDSBs despite the persistence of Ku or that alternatively, it displaces Ku when overexpressed. Future study will be required to test these hypotheses.

RAD52 acts as a mediator of ssDNA/RAD51 filaments during seDSB repair by HR in human cells. The severe TMZ sensitivity induced by loss of RAD52 and its strong ability to rescue the defective phenotypes associated with XAB2 depletion suggest that RAD52 also exerts a function independently of RAD51, in line with a previous report (14). Supporting this notion, we found that not all DSBs seen in XAB2-depleted cells are substrates for RAD51. RAD52 mediates MiDAS independently of RAD51 (15). It is therefore possible that the residual DSBs observed at 72 and 96 h in XAB2-depleted cells exposed to TMZ, which RAD52 overexpression fully suppressed, reflect in part the participation of XAB2 in MiDAS.

RAD52 inactivation induces synthetic lethality in cells harbouring deficiencies in key HR factors (95–98). Our observation that XAB2 limits the engagement of NHEJ associated with genome instability can explain why XAB2 was identified in RNAi screens for determinants of PARP inhibitor sensitivity (99,100) or for genes that promote genome stability (101). Using super-resolution imaging to examine the dynamics of HR proteins at CPT-induced seDSBs, Whelan *et al.* (13) have shown that, in human cells, the assembly of ssDNA/RAD51 nucleoprotein filaments is mediated by RAD52 or, in the absence of RAD52, by BRCA2. We reason that in the absence of RAD52, BRCA2 could promote RAD51 filament formation on resected seDSBs as well as RAD51-mediated BIR. However, RAD52-mediated BIR (including MiDAS) as well as other DSB repair pathways such as SSA would be non-functional. Unlike RAD51, our observations suggest that RAD52 may still promote some seDSB repair in the absence of XAB2. Whether BRCA2 is able to fulfil its RAD51-promoting role on resected seDSBs generated in the absence of XAB2 warrants further investigation as inept BRCA2 could contribute to the synthetic lethality observed in the absence of RAD52 and XAB2. However, it is likely that the observed lethality results in great part from defects in redundant as well as non-redundant DNA repair pathways involving XAB2 and RAD52. Our data suggest that strategies targeting XAB2 and/or RAD52 may help improve the therapeutic outcome of cancer patients treated with seDSBs-inducing DNA damaging agents. As GBM stem-like cells display high levels of DNA replication stress driving constitutive DDR activation and radiation resistance (102), targeting XAB2 and/or RAD52 may also benefit radiation-based strategies for GBM.

Although there is accumulating evidence that RNA splicing factors participate in DNA-damage repair (31–33,103,104), the underlying molecular mechanisms remain elusive. Unlike PRP19, XAB2 did not interact with RPA32 and its depletion did not prevent CPT-induced pS4-RPA32 foci formation. However, like PRP19, XAB2 was converted from a pre-mRNA splicing factor into a HR-promoting factor upon DNA damage, was required for efficient replication fork progression on damaged DNA and appeared to play a post-resection role during seDSB repair. Maréchal et al have discussed the possibility that PRP19, through its ability to associate with the transcription machinery and recognize RPA-coated ssDNA, functionally links DDR, transcription, and RNA processing (75). As our data suggest that XAB2 promotes processing events at the RPA-ssDNA platform upon replication fork collapse, and XAB2 has recently been involved in R-loop processing during development (89), we propose that the recognition and/or processing of RPA-coated ssDNA generated during DNA or RNA transactions represent a common feature of the splicing factors that promote DNA repair.

## Supplementary Material

gkab785_Supplemental_File
