# Supplementary material for: XAB2 promotes Ku eviction from single-ended DNA double-strand breaks independently of the ATM kinase
Source: Nucleic Acids Res. 2021 Sep 9;49(17):9906–25. doi: 10.1093/nar/gkab785 (PMC8464071; doi:10.1093/nar/gkab785)
Supplement: gkab785_Supplemental_File [file gkab785_supplemental_file.pdf]

Sharma et al. Supplementary Figures and Legends

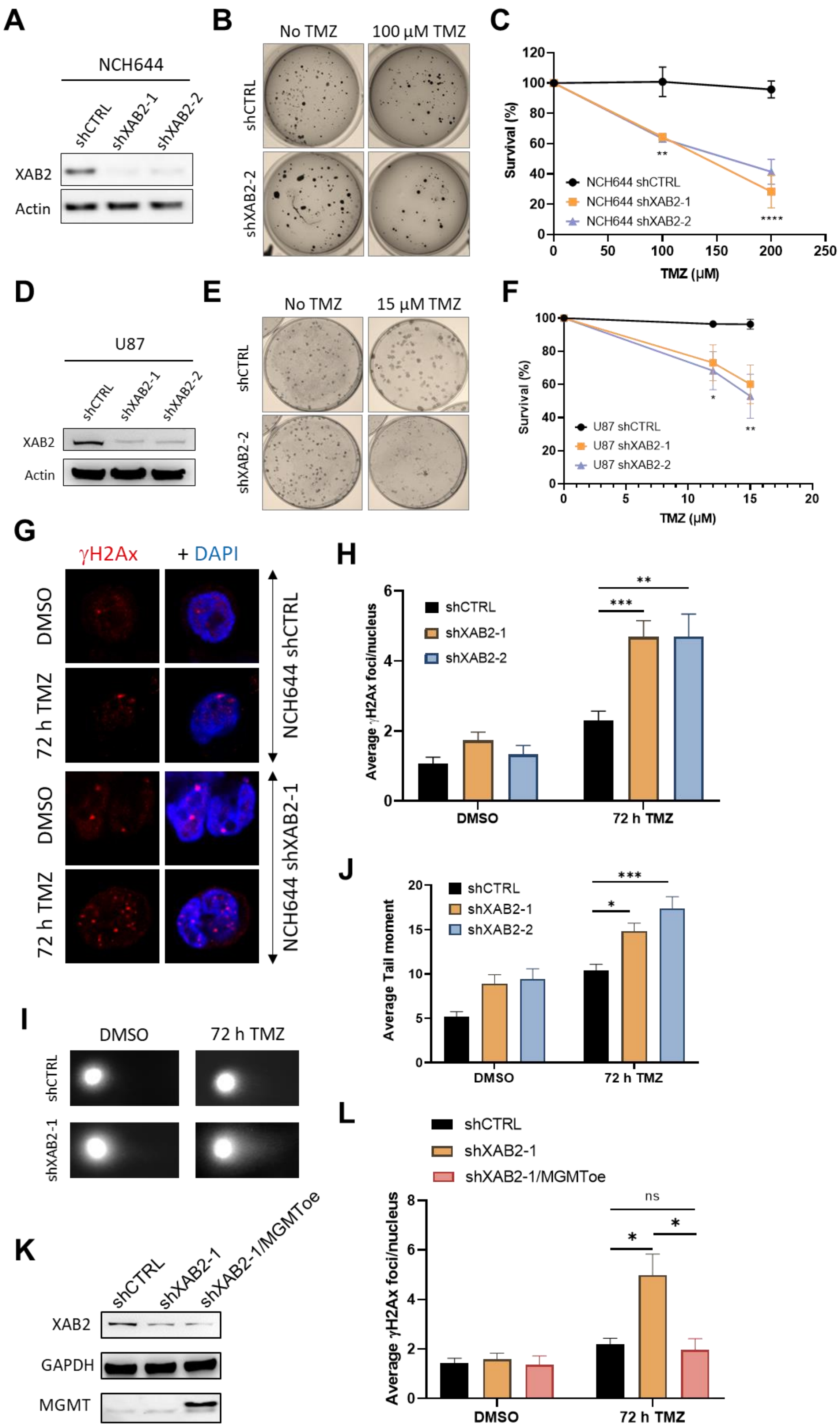
A
NCH644
shCTRL
shXAB2-1
shXAB2-2
XAB2
Actin
B
No TMZ
100 µM TMZ
shCTRL
shXAB2-2
C
Survival (%)
NCH644 shCTRL
NCH644 shXAB2-1
NCH644 shXAB2-2
TMZ (µM)
D
U87
XAB2
Actin
E
No TMZ
15 µM TMZ
F
U87 shCTRL
U87 shXAB2-1
U87 shXAB2-2
G
γH2Ax
+ DAPI
DMSO
72 h TMZ
NCH644 shCTRL
NCH644 shXAB2-1
H
Average γH2Ax foci/nucleus
I
J
Average Tail moment
K
shXAB2-1/MGMToe
GAPDH
MGMT
L
ns

Supplementary Figure 1. XAB2 is important for the repair of O^6-meG lesions induced by TMZ and left unrepaired by MGMT in NCH644 cells. (**A**-**F**) XAB2 depletion results in increased sensitivity to TMZ in NCH644 cells (**A**-**C**) and U87 cells (**D**-**F**). (**A** and **D**) Representative immunoblot illustrating the efficiency of XAB2 depletion achieved by 2 independent shRNAs (shXAB2-1 and shXAB2-2) as compared to a control, non-silencing shRNA (shCTL). Actin was used as a loading control. (**B** and **E**) Clonogenic survival assays. Cells were exposed to the indicated concentrations of TMZ or vehicle (DMSO) for 2 h and allowed to form colonies before being stained with crystal violet. (**C** and **F**) Quantification of the clonogenic assays following cell counting with ImageJ. Data are the average of n=3 or more biological replicates. Bars represent mean ± s.e.m. Significant differences between specified comparisons were assessed by one-way ANOVA, and highlighted by stars (*P<0.05; **P<0.01; ****P<0.0001). (**G**-**J**) XAB2 depletion impairs the repair of DSBs induced by unrepaired O6-meG. Representative immunofluorescence images of γH2AX foci (red) in NCH644 cells expressing the indicated shRNAs, following a 2 h exposure to 100 µM TMZ (or vehicle) and a 72 h recovery period (**G**), and related quantification (**H**). DNA was counterstained with DAPI (blue). (**I**-**J**) Comet assays. Control and XAB2-depleted NCH644 cells were treated with 100 µM TMZ (or vehicle) for 2 h and DNA damage was assessed by neutral comet assay after 72 h. Shown are representative images (**I**), and related quantification of the average mean tail moments (30 cell/sample/experiment (n=3) (**J**). (**K** and **L**) Ectopic MGMT expression prevents accumulation of TMZ associated seDSBs. (**K**) Immunoblot analysis of XAB2 and MGMT expression in NCH644 cells expressing shCTRL or shXAB2-1, as well as shXAB2-1-cells overexpressing MGMT (MGMToe). GAPDH was used as a loading control. (**L**) Quantification of the average number of γH2Ax foci per nucleus in the indicated NCH644 cells. Error bar ± s.e.m. Differences between specified comparisons were assessed by a

Mann-Whitney test and their significance is highlighted by stars (*P<0.05)(ns=non significant).

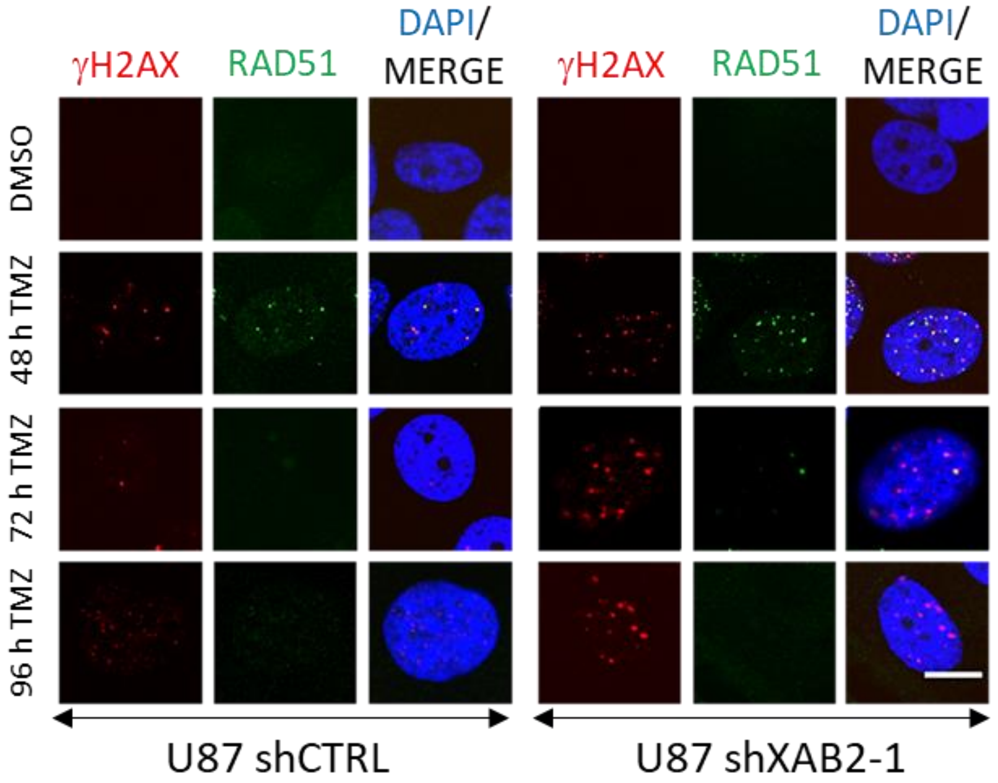

Supplementary Figure 2. Representative immunofluorescence images of RAD51 foci (green) in control and XAB2-depleted cells exposed to 15 μM TMZ (or DMSO) for 2 h and allowed to recover in drug-free medium for the indicated times. Also stained are γH2AX foci (red). See Figure 2D for related quantification. Scale bar: 5μm. The images are representative of 3 or more independent biological repeats.

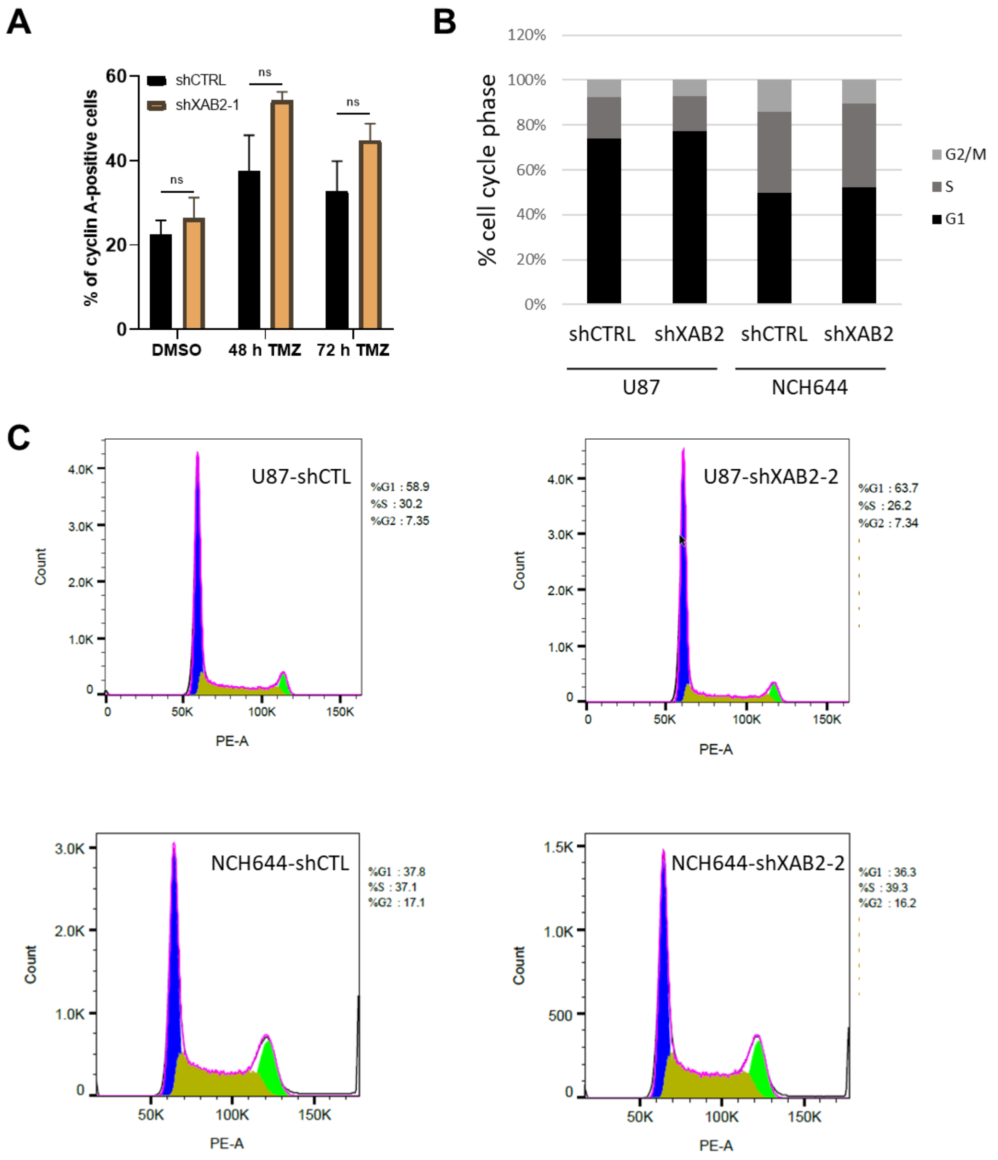

Supplementary Figure 3. XAB2 depletion does not affect cell cycle phase distribution. (**A**) U87 cells expressing the indicated shRNAs were treated for 2 h with 15µM TMZ (or DMSO) and left to recover for 48 h or 72 h in drug-free medium before being processed for IF analysis of Cyclin A. The graph presents the percentage of Cyclin A-positive cells in the indicated conditions. Error bar ± s.e.m. Differences between specified comparisons were assessed by a Mann-Whitney test and their significance is highlighted by stars (*P<0.05; **P<0.01; ***P<0.001)(ns = non significant). (**B** and **C**) Flow cytometry analysis. Shown are the percentage of cells in G1, S and G2/M based on propidium iodide staining of control and XAB2-depleted cells (**B**) and representative histograms (**C**) (N = 3).

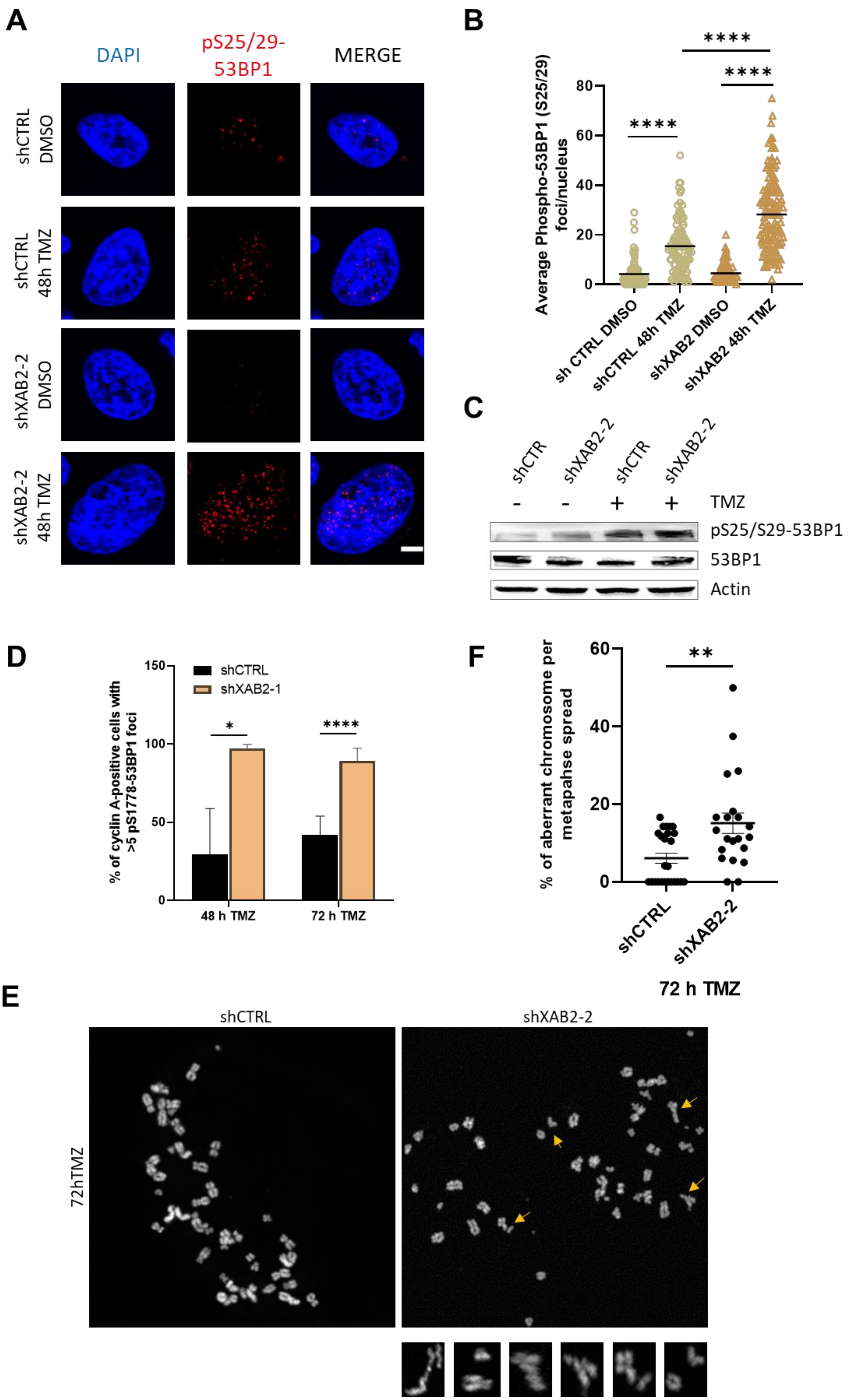
A
DAPI
pS25/29-53BP1
MERGE
shCTRL DMSO
shCTRL 48h TMZ
shXAB2-2 DMSO
shXAB2-2 48h TMZ
B
Average Phospho-53BP1 (S25/29) foci/nucleus
0
20
40
60
80
****
****
****
sh CTRL DMSO
shCTRL 48h TMZ
shXAB2 DMSO
shXAB2 48h TMZ
C
shCTR
shXAB2-2
shCTR
shXAB2-2
- - + + TMZ
pS25/S29-53BP1
53BP1
Actin
D
% of cyclin A-positive cells with >5 pS1778-53BP1 foci
150
100
50
0
shCTRL
shXAB2-1
*
****
48 h TMZ
72 h TMZ
F
% of aberrant chromosome per metapahse spread
60
40
20
0
**
shCTRL
shXAB2-2
72 h TMZ
E
shCTRL
shXAB2-2
72hTMZ

Supplementary Figure 4. (**A** and **B**) Representative immunofluorescence images of control and XAB2-depleted U87 cells following exposure to 15 μM TMZ (or DMSO) for 2 h, 48 h recovery in drug-free medium and processing for IF analysis of pS25/29-53BP1 foci (**A**) and related quantification of the average number of pS25/29-53BP1 foci per nucleus in the total cell population (**B**). Scale bar: 5µm. The images are representative of 3 independent biological repeats. Bars represent mean ± s.e.m. Significant differences between specified comparisons were assessed by one-way ANOVA and are highlighted by stars (****P<0.0001). (**C**) Western blot analysis of 53BP1 and pS25/29-53BP1 in control and XAB2-depleted U87 cells treated with TMZ or DMSO as in (**A**). XAB2 depletion did not affect 53BP1 protein expression levels but resulted in a 1.4-fold increase in 53BP1 phosphorylation following exposure to TMZ, compared to control cells. (**D**) Quantification of the percentage of Cyclin A-positive cells with > 5 pS1778-53BP1 foci per nucleus in control and XAB2-depleted U87 cells exposed to 15 μM TMZ (or DMSO) for 2 h before being allowed to recover in drug-free medium for the indicated times. Bars represent mean ± s.e.m. Significant differences between specified comparisons were assessed by 2-ways ANOVA and are highlighted by stars (*P<0.05; ****P<0.0001). (**E** and **F**) TMZ induces increased chromosomal aberrations in XAB2-depleted cells. U87 cells with the indicated shRNAs were exposed to 15µM TMZ (or DMSO) for 2 h and left to recover in drug-free medium for 72 h, followed by metaphase spread preparation and microscopic analysis of chromosomal aberrations (**E**) and their quantification (**F**). Yellow arrows indicate chromosomes for which enlarged images are presented in the lowest panel. Data are the average of n=2 biological replicates (30-50 cells/sample/experiment). Error bar ± s.e.m. Differences between samples were assessed by Mann-Whitney test and their significance is highlighted by stars (**P<0.01).

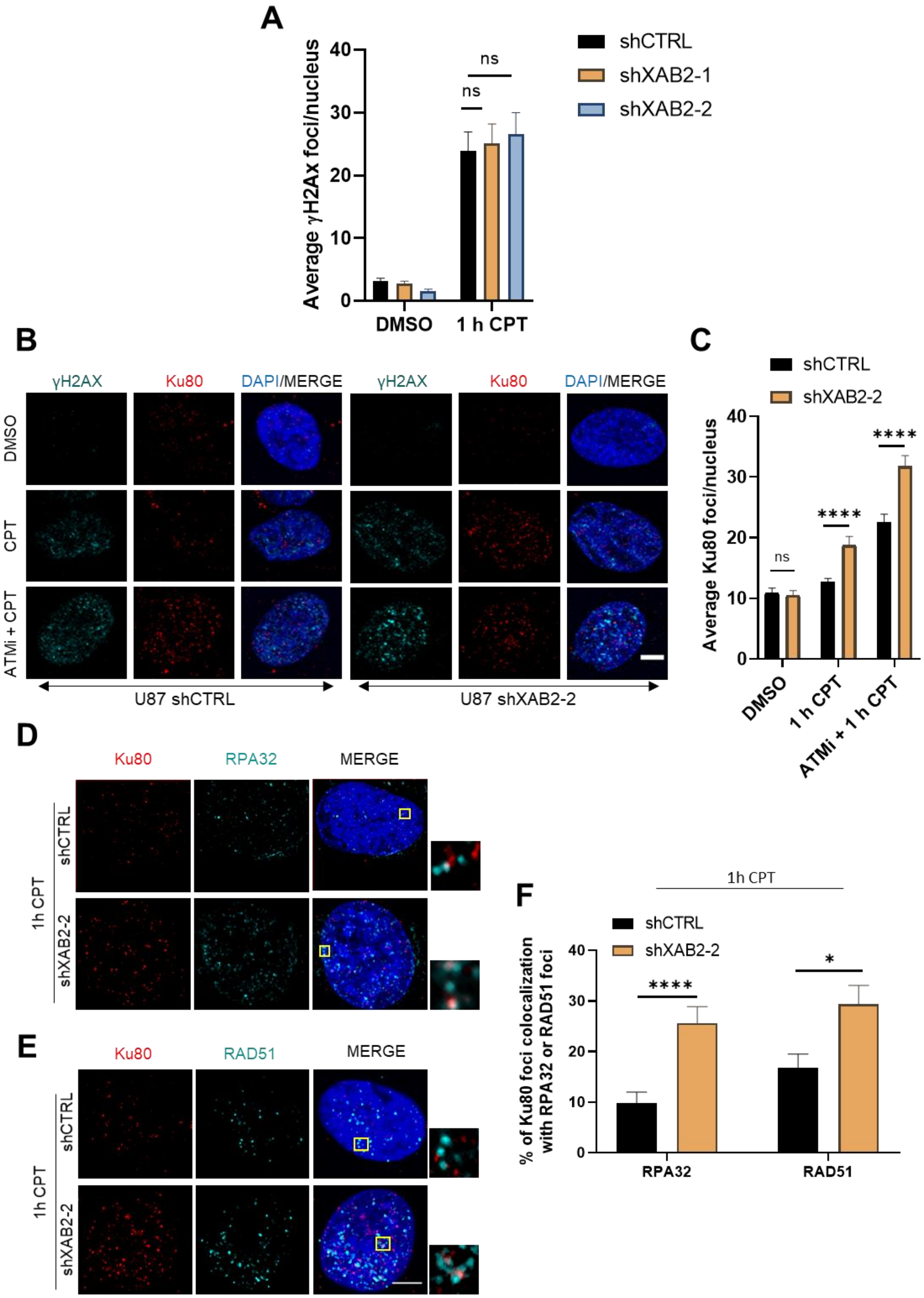
A
Average γH2Ax foci/nucleus
40
30
20
10
0
ns
ns
DMSO
1 h CPT
shCTRL
shXAB2-1
shXAB2-2
B
γH2AX
Ku80
DAPI/MERGE
γH2AX
Ku80
DAPI/MERGE
DMSO
CPT
ATMi + CPT
U87 shCTRL
U87 shXAB2-2
C
shCTRL
shXAB2-2
Average Ku80 foci/nucleus
40
30
20
10
0
ns
****
****
DMSO
1 h CPT
ATMi + 1 h CPT
D
Ku80
RPA32
MERGE
1h CPT
shCTRL
shXAB2-2
E
Ku80
RAD51
MERGE
1h CPT
shCTRL
shXAB2-2
F
1h CPT
shCTRL
shXAB2-2
% of Ku80 foci colocalization with RPA32 or RAD51 foci
40
30
20
10
0
****
*
RPA32
RAD51

Supplementary Figure 5. XAB2 prevents Ku retention on seDSBs induced by campthotecin. (**A**) Graph representing the average number of γH2Ax foci per nucleus in U87 cells expressing the indicated shRNAs, following exposure to 1 µM CPT for 1 h and processing for IF. Error bar ± s.e.m. Differences between samples were assessed by Mann-Whitney test and their significance is highlighted by stars (ns = non-significant). (**B** and **C**) Representative images of Ku80 foci (red) detected by immunofluorescence microscopy in control and XAB2-depleted cells exposed to 1 µM CPT for 1 h in the absence or presence of the ATMi KU-55933 (**B**) and related quantification (**C**). Cells were pre-treated with the ATMi or DMSO for 1 h prior to addition of CPT. Cells were also stained for γH2AX (cyan) and DNA was counterstained with DAPI (blue). Error bar ± s.e.m. Differences between samples treated with different drugs were assessed by Mann-Whitney test and their significance is highlighted by stars (****P<0.0001) (ns = non-significant). (**D**) Representative immunofluorescence images used for the quantification of the number colocalized Ku80 (red) and RPA32 (cyan) foci in individual cells, following exposure of control and XAB2-depleted to 1 µM CPT for 1 h. The rightmost images present close up sections (rectangles) illustrating the extensive colocalization of RPA and Ku80 foci in XAB2-depleted cells compared to control cells. (**E**) Same as in (**D**) for the analysis of Ku80 (red) and RAD51 (cyan) foci colocalization. (**F**) Quantification of the frequencies of colocalized Ku80-RPA32 foci and Ku80-RAD51 foci in control and XAB2-depleted cells exposed to CPT, as assessed based on (**D**) and (**E**). Scale bar= 5µm. Data are the average of n=3 biological replicates (30-50 cells/sample/experiment). Error bar ± s.e.m. Differences between samples were assessed by Mann-Whitney test and their significance is highlighted by stars (****P<0.0001) (ns = non-significant).

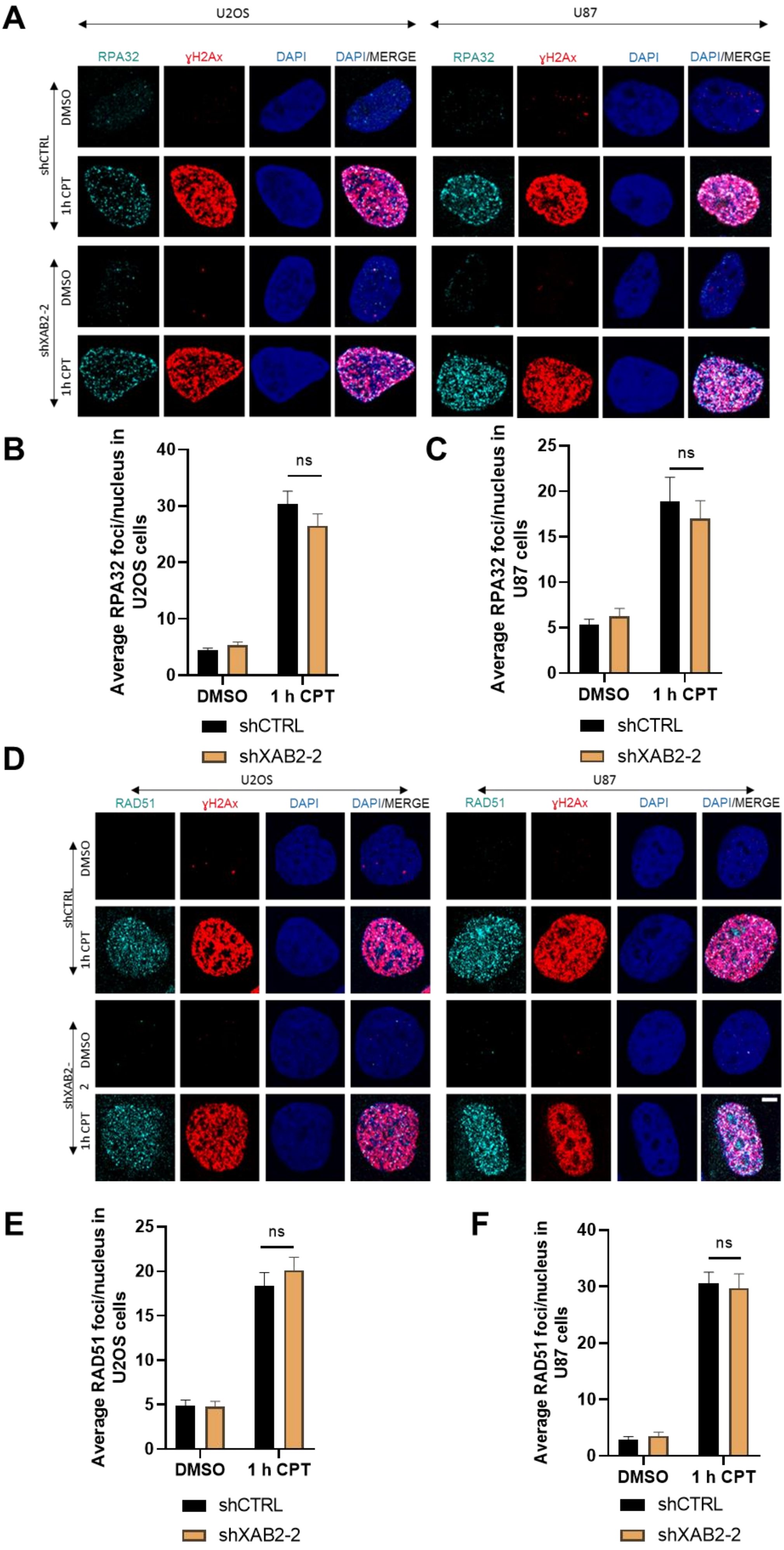
A
U2OS
U87
RPA32
γH2Ax
DAPI
DAPI/MERGE
shCTRL
DMSO
1h CPT
shXAB2-2
B
Average RPA32 foci/nucleus in U2OS cells
40
30
20
10
0
ns
DMSO
1 h CPT
shCTRL
shXAB2-2
C
Average RPA32 foci/nucleus in U87 cells
25
20
15
10
5
0
D
RAD51
E
Average RAD51 foci/nucleus in U2OS cells
F
Average RAD51 foci/nucleus in U87 cells

Supplementary Figure 6. XAB2 depletion does not affect RPA32 and RAD51 foci formation in U2OS and U87 cells treated with CPT. (**A**-**C**) Representative immunofluorescence images of RPA32 (cyan) and γH2AX (red) foci in U2OS and U87 cells expressing shCTRL or shXAB2-2, following exposure to 1 μM CPT (or DMSO) for 1 h (**A**) and related quantification of RPA32 foci in U2OS (**B**) and U87 (**C**). (**D**-**F**) Same as (**A**-**C**) for the analysis of RAD51 foci (cyan). Scale bar=5μm. Data are the average of n>2 biological replicates (30-50 cells/sample/experiment). Error bar ± s.e.m. Differences between samples treated with different drugs were assessed by Mann-Whitney test and their significance is highlighted by stars or ns (non-significant).

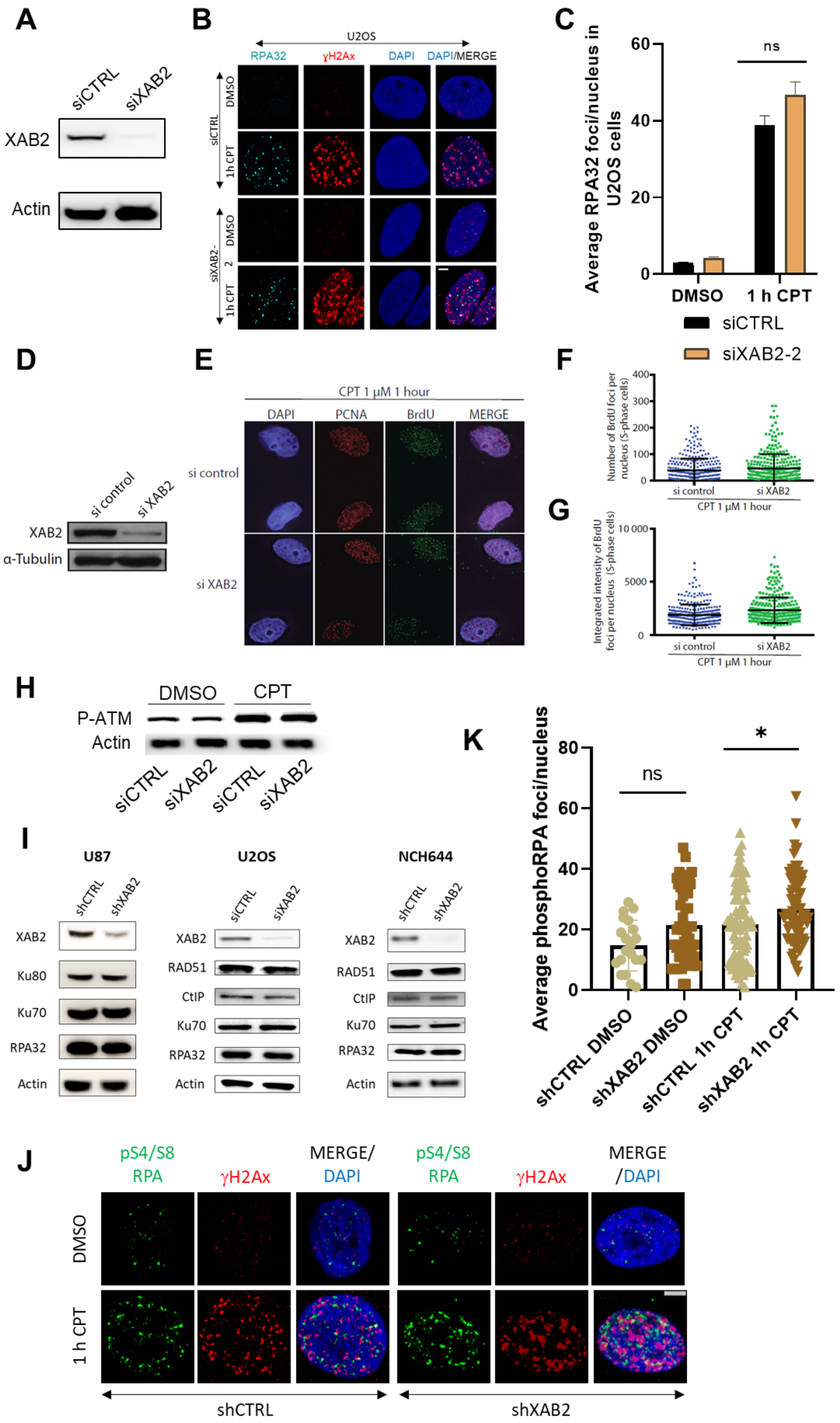
A
siCTRL
siXAB2
XAB2
Actin
B
U2OS
RPA32
γH2Ax
DAPI
DAPI/MERGE
siCTRL
DMSO
1h CPT
siXAB2-2
DMSO
1h CPT
C
Average RPA32 foci/nucleus in U2OS cells
60
40
20
0
ns
DMSO
1 h CPT
siCTRL
siXAB2-2
D
si control
si XAB2
XAB2
α-Tubulin
E
CPT 1 μM 1 hour
DAPI
PCNA
BrdU
MERGE
si control
si XAB2
F
Number of BrdU foci per nucleus (S-phase cells)
400
300
200
100
0
si control
si XAB2
CPT 1 μM 1 hour
G
Integrated intensity of BrdU foci per nucleus (S-phase cells)
10 000
5000
0
si control
si XAB2
CPT 1 μM 1 hour
H
DMSO
CPT
P-ATM
Actin
siCTRL
siXAB2
siCTRL
siXAB2
I
U87
shCTRL
shXAB2
XAB2
Ku80
Ku70
RPA32
Actin
U2OS
siCTRL
siXAB2
XAB2
RAD51
CtIP
Ku70
RPA32
Actin
NCH644
shCTRL
shXAB2
XAB2
RAD51
CtIP
Ku70
RPA32
Actin
K
Average phosphoRPA foci/nucleus
80
60
40
20
0
ns
*
shCTRL DMSO
shXAB2 DMSO
shCTRL 1h CPT
shXAB2 1h CPT
J
pS4/S8 RPA
γH2Ax
MERGE/DAPI
pS4/S8 RPA
γH2Ax
MERGE /DAPI
DMSO
1 h CPT
shCTRL
shXAB2

Supplementary Figure 7. (**A**-**C**) Analysis of RPA foci. (**A**) Immunoblotting analysis of XAB2 depletion in U2OS cells treated with non-silencing siRNAs (siCTRL) or siRNAs targeting XAB2 (siXAB2). Actin was used as loading control. (**B** and **C**) Representative immunofluorescence images of RPA32 (cyan) and gH2AX (red) foci in U2OS transfected with siCTRL or siXAB2 following exposure to 1 μM CPT (or DMSO) for 1 h (**B**), and related quantification of RPA32 foci (**C**). Scale bar= 5µm. Data are the average of n=3 biological replicates (30-50 cells/sample/experiment). Error bar ± s.e.m. Differences between samples treated with different drugs were assessed by Mann-Whitney test and their significance is highlighted by stars (ns = non-significant). (**D**-**F**) Native BrdU assay to detect ssDNA. (**D**) Western blotting to validate the knockdown of XAB2 in U2OS cells used in the assay. (**E**) U2OS cells transfected with a control or XAB2 siRNA were incubated with BrdU for 24 h followed by treatment with 1 µM CPT for 1 h and a 1 h release period. The cells were then subjected to an immunofluorescence analysis against bromodeoxyuridine (BrdU) and PCNA. (**F**) Quantification of the number of foci. The data show the mean ± s.d. The difference between the two conditions is not significant (Mann-Whitney U-test). (**G**) Quantification of the integrated intensity of BrdU foci per nucleus. The data show the mean ± s.d. **** $p \leq 0.0001$ (Mann-Whitney U-test). (**H**) Immunoblotting analysis of P-ATM in extracts from U2OS cells transfected with siCTRL or siXAB2, following treatment with CPT. (**I**) Western blot analysis of the indicated DNA repair factors in control or XAB2-depleted U87, U2OS and NCH644 cells. (**J** and **K**) Representative immunofluorescence images of pS4/S8-RPA32 (green) and γH2AX (red) foci in control and XAB2-depleted U87 cells, following exposure to 1 μM CPT (or DMSO) for 1 h (**J**) and related quantification of pS4/S8-RPA32 foci in U2OS (**K**). Data are representative of 2 independent biological repeats. Bars represent mean ± s.e.m. Significant differences between specified comparisons were assessed by one-way ANOVA (*$P<0.05$; ns=not significant).

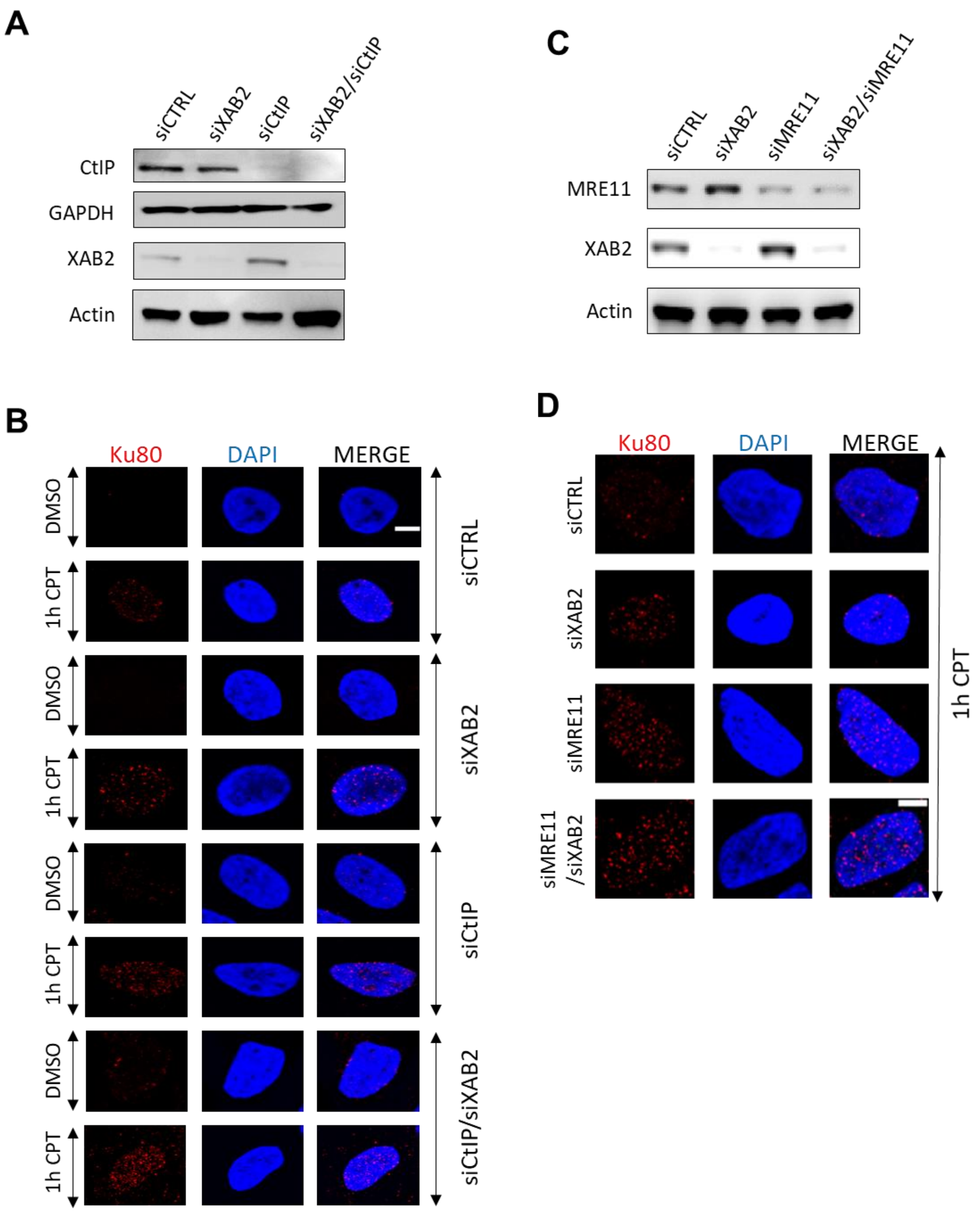
A
siCTRL
siXAB2
siCtIP
siXAB2/siCtIP
CtIP
GAPDH
XAB2
Actin
C
siCTRL
siXAB2
siMRE11
siXAB2/siMRE11
MRE11
XAB2
Actin
B
Ku80
DAPI
MERGE
DMSO
1h CPT
siCTRL
siXAB2
siCtIP
siCtIP/siXAB2
D
Ku80
DAPI
MERGE
siCTRL
siXAB2
siMRE11
siMRE11
/siXAB2
1h CPT

Supplementary Figure 8. (**A** and **C**) Immunoblotting analysis of XAB2 and/or CtIP depletion (**A**) or XAB2 and/or MRE11 depletion (**C**) in U2OS cells treated with non-silencing siRNAs (siCTRL) or siRNAs targeting XAB2, CtIP or MRE11. Actin was used as loading control. (**B** and **D**), Representative images of Ku80 foci (red) detected by IF microscopy in cells transfected with the indicated siRNAs and exposed to DMSO or 1 μM CPT for 1 h. See Figure 4A,B for quantification. Scale bar=5μm.
